# Cost and economic evidence of stroke technology-assisted rehabilitation: a scoping review

**DOI:** 10.1186/s12913-026-15267-7

**Published:** 2026-08-11

**Authors:** Tengiz Samkharadze, Olivier Lambercy, Roger Gassert, Karen Sui Geok Chua, David Bruce Matchar, Simone Kager, Nicole Wenderoth, Hsiao-ju Cheng

**Affiliations:** 1 Singapore-ETH Centre, Future Health Technologies Programme, CREATE Campus, Singapore, 138602 Singapore; 2 Rehabilitation Engineering Laboratory, Department of Health Sciences and Technology, ETH Zurich, Zurich, 8092 Switzerland; 3 Rehabilitation Research Institute of Singapore, Nanyang Technological University, Singapore, 308232 Singapore; 4 Institute of Rehabilitation Excellence, Tan Tock Seng Hospital Rehabilitation Centre, Singapore, 307382 Singapore; 5 Yong Loo Lin School of Medicine, National University of Singapore, Singapore, 117597 Singapore; 6 Lee Kong Chian School of Medicine, Nanyang Technological University, Singapore, 308232 Singapore; 7 Programme in Health Services and Systems Research, Duke-NUS Medical School, National University of Singapore, Singapore, 169857 Singapore; 8 Department of Internal Medicine (General Internal Medicine), Duke University Medical School, Durham, NC 27710 USA; 9 Department of Computer Science and Artificial Intelligence, University of Technology Nuremberg, 90461 Nuremberg, Germany; 10 Neural Control of Movement Laboratory, Department of Health Sciences and Technology, ETH Zurich, Zurich, 8092 Switzerland

**Keywords:** Economic cost, Effectiveness, Rehabilitation, Stroke, Technology

## Abstract

**Introduction:**

In recent years, the growing demand for cost-effective rehabilitation after stroke has motivated the development of novel technologies to support high-intensity rehabilitation. However, available knowledge on the economic benefits of stroke technology-assisted rehabilitation (TAR) is relatively scarce and fragmented. Therefore, this scoping review aimed to review evidence on the economic benefits of TAR in laboratory, clinical, and home settings, as well as the methodologies used to evaluate these outcomes.

**Methods:**

A systematic search of four electronic bibliographic databases and two additional grey literature/ economic evidence sources (until 03 June 2026) identified 6469 records. Following predefined inclusion and exclusion criteria, 44 records were considered for further analysis. Additionally, 21 records were identified through a citation search.

**Results:**

In total, 65 articles reporting TAR-related costs were included, but only a few of these studies reported cost analyses (mostly cost comparisons). Firstly, of the 38 studies conducted in laboratory settings, only one reported a formal TAR-related cost analysis. Secondly, out of 16 studies reporting costs, 11 studies performed cost analysis for TAR in clinical settings. Of these, the majority (six) estimated TAR to be equally effective or even superior (one) and cheaper than conventional therapy (CT) in the clinic, but only when TAR enabled the therapist to treat several stroke survivors in parallel. This was captured using the relative therapist-to-patient ratio (rT/P), indicating that staffing and supervision models were more important than device cost. Lastly, seven of 13 studies conducted TAR-related cost analysis in a home setting. Three studies suggested that TAR may be economically beneficial compared with CT in the clinic.

**Conclusion:**

Empirical findings suggest that TAR may generate cost savings compared to CT in the clinic, particularly when rT/P is < 0.5. However, the current evidence is sparse, with limited comparability and generalisability due to heterogeneous TAR modalities, comparators and cost-calculation methods.

**Clinical trial number:**

Not applicable.

**Supplementary Information:**

The online version contains supplementary material available at https://doi.org/10.1186/s12913-026-15267-7.

## Introduction

Stroke is the third leading cause of death and disability worldwide [1]. The estimated stroke-related healthcare expenditure is 1.12% (891 billion US$) of the global gross domestic product [1]. It is predicted that the stroke-related health-economic burden will remain high or further increase in high and low- to middle-income countries, respectively, mainly due to the rapidly ageing population and high exposure to behavioural risk factors (i.e., dietary habits, poorly managed non-communicable cardio-vascular diseases) [1, 2]. Annually, 12.2 million new cases of stroke are recorded globally and these numbers are expected to rise due to demographic changes. Of these, 62% occur among adults aged 50 to 70 years [1]. As a result of the growing number of stroke survivors, healthcare systems face a high demand for services, including conventional rehabilitation therapy (CT), which should be widely accessible and cost-effective. In our study, CT refers to therapist-led rehabilitation, which may include physiotherapy, occupational therapy, task-specific training, or other standard rehabilitation programmes. Although CT varies across health systems in service delivery, intensity, staffing models, and reimbursement, it serves as a practical reference for evidence mapping by reflecting rehabilitation pathways that are considered standard of care [3] and can be supplemented, substituted, or augmented in clinical practice.

Adequate rehabilitation therapy is key to increasing post-stroke functional recovery rates and reducing disability-caused economic burden [2, 4, 5]. Approximately 67% of stroke survivors, after their health condition is stabilised, require intensive rehabilitation therapy targeting sensorimotor functions [5]. However, access to sufficiently dosed rehabilitation is limited due to a shortage of healthcare personnel [2, 6, 7] and high hospital care costs (8–12% of total care in a clinic) [8, 9].

In recent years, a new concept of stroke Technology-Assisted Rehabilitation (TAR) has been proposed as an alternative to CT models [5, 6, 10, 11]. TAR is an umbrella term for rehabilitation devices, systems, and platforms that primarily support the delivery of sensorimotor training after stroke [5]. This broad category includes technologies designed for a particular therapy context (i.e., clinic, home) [10] and for a specific therapy target (i.e., upper or lower limb function) [11–13]. Given the heterogeneity of TAR devices in terms of mechanisms of action, cost structure, supervision requirements, and implementation pathways, devices targeting motor function can be pragmatically categorised in two broad groups, actuated or non-actuated, to facilitate evidence mapping. Actuated devices, which incorporate motors to support movement execution, include systems such as end-effector robots, actuated soft robotics devices and exoskeletons. In contrast, non-actuated devices do not use powered actuation but support therapy through feedback, guidance, monitoring or mechanical assistance, using virtual reality, wearable sensors, brain-computer interfaces (BCIs), muscle-computer interfaces (MCIs), non-actuated braces, gravity-supported orthoses, spring-based systems or camera systems.

The expected advantages of integrating TAR concepts into clinical practice include increased intensity of task-specific sensorimotor training, greater reproducibility, and objective quantification and monitoring of training outcomes. These aspects are likely to benefit stroke survivors by increasing the dose, quality of the rehabilitation training or personalisation of the therapy plan and, thereby, potentially increasing recovery gains relative to CT [10, 11]. Technologies that enable semi- or minimally supervised training regimes can additionally reduce manpower demand, for example, by facilitating group sessions in clinics. Such concepts are likely to increase access to therapy despite the shortage of therapists [5, 10, 14]. With remote monitoring, minimally or unsupervised TAR could allow stroke survivors to continue training in community centres or at their homes [5, 10, 11]. Thereby, TAR eliminates significant functional and social barriers that might prevent stroke survivors from participating in rehabilitation training [10].

From the economic perspective, it has been hypothesised that applying minimally or unsupervised TAR could save healthcare costs compared to existing CT models [14–17]. As less labour-intensive models of care, semi- and unsupervised TAR services are likely to reduce costs related to therapists’ and caregivers’ work time, as well as stroke survivors’ leisure time. TAR delivered in home settings or in nearby community centres is likely to also save transportation time and transportation costs. However, in general, empirical evidence on TAR’s cost and its cost analysis is currently scarce [14–17]. Additionally, much of this evidence is generated using non-standardised cost analysis methods, which further restricts its comparability across different health systems [14–16]. Despite the mentioned limitations, previous review papers that synthesised evidence on TAR-related health-economic impact argue that technology could lead to economic benefits in comparison to CT if the devices reduce demand on therapist time while providing comparable clinical effectiveness [14–17]. Additionally, when unsupervised TAR can be performed in the home of the stroke survivors, in addition to direct healthcare costs, transportation-related costs could be saved for severely impaired individuals [16]. However, those review papers primarily focus on individual technology categories (i.e., robotic rehabilitation, virtual reality), rather than examining how economic evidence is generated and reported across different TAR modalities, stages of technology development, and deployment.

This scoping review aims to provide a comprehensive overview of cost and economic evidence across all levels of supervision, including fully supervised, semi-supervised, and unsupervised TAR models, spanning from laboratory prototypes to clinical and home-based applications. Rather than concentrating on specific technology categories, our primary objective was to map how economic evidence has been generated within the TAR field. This broader approach allows us to identify common cost structures, economic evaluation methods, and existing methodological gaps across otherwise-separated technology categories and service delivery pathways. To support evidence mapping, TAR device types were broadly classified as actuated and non-actuated, with a focus on neuro-motor rehabilitation.

## Methods

### Search strategy

A systematic search was performed in the four electronic databases: PubMed (Central), Embase, IEEE Xplore and Cochrane Database of Systematic Reviews (Central). To identify relevant grey literature/economic evidence, the International Health Technology Assessment (INAHTA) and Cost-Effectiveness Analysis (CEA) registries were searched. In addition, we used a citation search to identify other relevant records. We searched for stroke, upper or lower limb, technology-assisted rehabilitation, and related health-economic analysis. The initial search was performed on 12 September 2022, which was updated on 26 December 2023, 29 May 2025 and with additional keywords during 28 May-3 June 2026. Detailed keyword strings are provided in Appendix Table 1. Reference management was done in Zotero (version 6.0.18).

**Table 1:** Reported devices’ production cost or retail price across settings

Settings	Authors, Year	Study Type	Type of TAR Devices		Limb	Sample (Able-Bodied Individuals/ Stroke survivors, Mixed)	Sample Size (*n*)	TAR Device’s Production Cost or *Retail Price* (US$)
Laboratory Settings	Sarker A. et al., 2022 [28]	-	Non-actuated	Wearable sensors	U	Able-bodied	21	500
	Postolache O. et al., 2021 [29]	Case series	Non-actuated	VR & wearable sensors	U	Able-bodied	2	1900
	Nissler C. et al., 2019 [30]	Quasi-experiment	Non-actuated	VR & wearable sensors	U	Able-bodied	15	3545.1*
	McCrimmon C. M. et al., 2017 [31]	Case series	Non-actuated	BCI	U	Able-bodied	5	310
	Rinne P. et al., 2016 [32], 2	Quasi-experiment	Non-actuated	VR & sensors	U	Stroke survivors	87	600
	Seo N. J. et al., 2016 [33]	Case series	Non-actuated	VR & wearable sensors	U	Stroke survivors	10	160
	Nguyen K.D. et al., 2011 [34]	Case series	Non-actuated	Wearable sensors	U	Able-bodied	5	40
	Segal A.D. et al., 2020 [35]	Quasi-experiment	Non-actuated	Gesture controlled robot	U	Able-bodied	11	135
	Du Z. et al., 2026 [36]	Case series	Non-actuated	Exoskeleton	U	Able-bodied	8	20
	Hughes M.L.C. et al., 2019 [37]	Quasi-experiment	Non-actuated	Wearable sensors	U	Able-bodied	31	30
	Vourvopoulos A. et al., 2016 [38]	Quasi-experiment	Non-actuated	BCI	U	Able-bodied	8	234.1*
	Washabaugh E.P. et al., 2019 [39]	Case report	Non-actuated	Resistive robot	U	Able-bodied	1	3800
	McAmis S. et al., 2013 [40]	-	Non-actuated	Spring-based system	U	Able-bodied	6	700
	Washabaugh E.P. et al., 2018 [41]	Quasi-experiment	Non-actuated	Resistive robot	L	Stroke survivors	6	2000
	Khoo I-H. et al., 2017 [91], 1	Case series	Non-actuated	Wearable sensors	L	Able-bodied	6	500
	Ates S. et al., 2013 [92], 4	Quasi-experiment	Non-actuated	Spring-based exoskeleton & VR	U	NR	NR	250
	Kim J. et al., 2025 [42]	Case series	Non-actuated	End-effector robot	U	Able-bodied	15	130
	Saldanha D.J. et al., 2026 [43]	Quasi-experiment	Non-actuated	Computer vision	L	Able-bodied	8	1056*
	Redd C.B. et al., 2012 [44]	Quasi-experiment	Non-actuated	Wearable sensors	L	Able-bodied	12	225
	Cao S. et al., 2025 [45]	Quasi-experiment	Non-actuated	VR	L	Mixed	7	330
	Zedda A. et al., 2025 [46]	Quasi-experiment	Non-actuated	VR & wearable sensors	B	Able-bodied	11	2148.1*
	Placidi G. et al., 2013 [47]	Quasi-experiment	Non-actuated	VR	U	Able-bodied	10	500
	Araujo R.S. et al., 2021 [48], 3	Case report	Actuated	Exoskeleton & BCI	U	Able-bodied	1	125
	Johnson M.J. et al., 2017 [49]	Case series	Actuated	Robot gym *(1 device)*	B	Stroke survivors	4	5500
	Panny M. et al., 2020 [50]	-	Actuated	End-effector robot & VR	U	*NR*	*NR*	336*
	Koçak M. et al., 2022 [51]	Case report	Actuated	End-effector robot	U	Able-bodied	1	170.1
	Yurkewich A. et al., 2020 [52]	Quasi-experiment	Actuated	Exoskeleton	U	Stroke survivors	11	500
	Rudd G. et al., 2019 [53]	-	Actuated	Exoskeleton	U	*NR*	*NR*	200
	Jochumsen M. et al., 2021 [54]	Quasi-experiment	Actuated	Exoskeleton & BCI	U	Able-bodied	11	1000
	Ou Y-K. et al., 2020 [55]	-	Actuated	Exoskeleton	U	*NR*	*NR*	650
	Dwivedi A. et al., 2019 [56]	Case series	Actuated	Exoskeleton & MCI	U	NR	3	1000
	Ona Simbana E.D. et al., 2018 [57]	Case series	Actuated	End-effector robot	U	Mixed	9	1395*
	Zhao Y. et al., 2025 [58]	-	Actuated	Exoskeleton	U	*NR*	*NR*	1530
	Araujo A. et al., 2025 [59]	Quasi-experiment	Actuated	Exoskeleton & VR	U	Able-bodied	16	189.9*
	Zheng S. et al., 2025 [60]	-	Actuated	Exoskeleton & wearable sensor	U	NR	NR	148.7
	Gonçalves A. et al., 2026 [61]	-	Actuated	Exoskeleton	U	NR	NR	924.6*
	Payra S. et al., 2021 [62]	Case report	Actuated	Exoskeleton	L	Able-bodied	1	600
	Sanz-Pena I. et al., 2021 [63]	Case report	Actuated	Exoskeleton & MCI	L	Able-bodied	1	800
Clinical Settings	Bustamante Valles K. et al., 2016 [64]	RCT	Actuated	Robot gym	B	Chronic stroke	27	*36048*
	Hesse S. et al., 2014 [65]	RCT	Actuated	Robot gym	U	Subacute stroke	50	*47* 812*
	Pila O. et al., 2022 [66]	RCT + Cohort	Actuated	End-effector robot	U	Subacute stroke	36	107903.9*
	McCabe J. et al., 2015 [67]	RCT	Actuated	End-effector robot	U	Chronic stroke	39	*890* *00*
	Vanoglio F. et al., 2017 [68]	RCT	Actuated	Exoskeleton	U	Subacute stroke	30	26630.4*
	Fernandez-Garcia C. et al., 2021 [69, 93, 94]	RCT	Actuated	Robot gym	U	Subacute-Chronic stroke	770	*133* 6300*
	Lo A.C. et al. 2010 [70, 95]	RCT	Actuated	Robot gym	U	Chronic stroke	127	*23* *0750*
	Micera S. et al., 2005 [71]	Quasi-experiment	Actuated	End-effector robot	U	Chronic stroke	8	5534.1*
	Singh N. et al., 2019 [72]	Quasi-experiment	Actuated	Exoskeleton & MCI	U	Chronic stroke	12	550
	Jayavel P. et al., 2024 [73]	RCT	Actuated	Exoskeleton	U	Chronic stroke	20	182
	Masiero S. et al. 2014 [74, 96, 97]	RCT [96, 97]	Actuated	End-effector robot	U	Subacute stroke	34 [97] & 21 [96]	66405*
	Shankar R. et al., 2025 [75]	non-RCT	Actuated	Exoskeleton	L	Subacute stroke	62	*20* *9642*
	Batavia M. et al., 1997 [76]	Case report	Non-actuated	Sensor	Trunk	Subacute stroke	1	10
	Khoo I-H. et al., 2017 [91], 1	Quasi-experiment	Non-actuated	Wearable sensors	L	Chronic stroke	6	500
	Islam M. K. et al., 2019 [77, 98]	RCT	Non-actuated	VR & wearable sensors	U	Subacute stroke	120	*37720*
	Rémy-Néris O. et al., 2021 [78]	RCT	Non-actuated	Spring-based gravity supported exoskeleton & VR	U	Subacute stroke	215	*73220*
Home Settings	Fusari G. et al., 2025 [79]	RCT	Non-actuated	Wearable sensors	U	Stroke survivors	30	*4287* *
	Lloréns R. et al., 2015 [80]	RCT	Non-actuated	VR	L	Chronic stroke	31	800
	Veras M. et al., 2020 [81]	RCT	Non-actuated	VR	U	Chronic stroke	51	6071*
	Adie K. et al., 2017 [82]	RCT	Non-actuated	VR	U	Subacute-Chronic stroke	235	NR
	Flynn S.P.T. et al., 2007 [83]	Case report	Non-actuated	VR	U	Chronic stroke	1	200
	Marin-Pardo O. et al., 2021 [84]	Case report	Non-actuated	MCI (VR & sensor)	U	Chronic stroke	1	150
	Burgos P.I. et al., 2020 [85]	Quasi-experiment	Non-actuated	VR & sensors	L	Subacute stroke	10	410
	Smayda K.E. et al., 2024 [86, 99, 100]	Case series [99] & RCT [100]	Non-actuated	Wearable sensors & auditory feedback	L	Chronic stroke	15 [99] & 87 [100]	NR
	Ates S. et al., 2013 [92], 4	Quasi-experiment	Non-actuated	Spring-based Exoskeleton & VR	U	Chronic stroke	10	250
	Housley S.N. et al., 2016 [87]	Quasi-experiment	Actuated	End effector robots & VR	B	Subacute- Chronic	20	NR
	Aguirre-Ollinger G. et al., 2024 [88, 101]	Quasi-experiment [88] & RCT [101]	Actuated	End effector robot	U	Subacute-Chronic stroke	12 [88] & 41 [101]	NR
	Sivan M. et al., 2014 [89]	Quasi-experiment	Actuated	End effector robot	U	Chronic stroke	19	8400
	Pergami-Peries M. et al., 2025 [90]	Quasi-experiment	Actuated	Exoskeleton	U	Chronic stroke	14	350

Table 1

Retail prices are italicised

*Costs were converted from the original currency to US$

• Nissler C. et. al., 2019, Euro 1=US$ 1.1817 in 2018

• Vourvopoulos A. et al., 2016, Euro 1=US$ 1.1096 in 2015

• Panny M. et. al., 2020, Euro 1=US$ 1.1194 in 2019

• Ona Simbana E.D. et al., 2018, Euro 1=US$ 1.1817 in 2018

• Saldanha D.J. et al., 2026, Indian Rupee 1=US$ 0.0106 in Jan. 2026

• Zedda A. et al., 2025, Euro 1=US$ 1.1306 in 2025

• Araujo A. et al., 2025, Euro 1=US$ 1.1306 in 2025

• Gonçalves A. et al., 2026, Euroe 1=US$ 1.1557in March 2026

• Hesse S. et al., 2014, Euro 1=US$ 1.3281 in 2013

• Pila O. et al., 2022, Euro 1=US$ 1.1194 in 2019

• Vanoglio F. et al., 2017, Euro 1=US$ 1.1096 in 2016

• Rémy-Néris O. et al., 2021, Euro 1=US$ 1.1847 in 2021

• Fusari G. et al., 2025, GBP 1=US$ 1.3192 in Jan. 2025

• Fernandez-Garcia C. et al., 2021, GBP 1=US$ 1.3363 in 2018

• Masiero S. et al. 2014, Euro 1=US$ 1.3281 in 2014

• Veras M. et al., 2020, CA 1 in 2015

• Adie K. et al., 2017, GBP 1=US$ 1.5642 in 2013

• Aguirre-Ollinger G. et al., 2024, SG 1 in 2021

1. Khoo I-H. et al. reported technology validation in the laboratory and clinic

2. Rinne et al. reported the prototype cost, which combines the maximal price for the smartphone deviceand the developed system

3. Araujo R.S. et al. tested BCI & Exoskeleton in combination. However, the authors engineered only the exoskeleton and provided its cost

4. Ates S. et al. reported technology validation in the laboratory and at home

^a^ BCI=Brain-Computer Interface, B=Both Limbs, L=Lower Limb, MCI=Muscle-Computer Interface, NR=Not Reported, RCT^=^Randomized Control Trial, TAR= Stroke Technology Assisted Rehabilitation, U=Upper Limb, VR=Virtual Reality

### Inclusion/ exclusion criteria

We applied the following inclusion criteria to select relevant studies: (1) reported use of TAR in either laboratory, clinic, or home settings, (2) TAR designed for the adult population, operationalised as participants aged ≥ 19 and (3) reported TAR-related economic cost or cost analysis. The ≥ 19-year threshold was used as an operational eligibility criterion for the review and was informed by standard age classifications, considering the World Health Organisation’s classification of adolescence as 10–19 years [18]. The reports were excluded if the studies were (1) not published in English or (2) without a fully accessible text.

### Records screening and data extraction

All records were screened by the first reviewer (TS), and a random 10% subset was independently screened at the title/abstract and full-text stages by the second reviewer (H-JC), with the aim of achieving 90% consistency in applying the eligibility criteria [19]. Disagreements were resolved through discussion and, where necessary, through consultation with a third reviewer (NW).

Data extraction was performed by the first reviewer using a pre-defined charting form (see below respective sections). A subset of the included records was independently checked by the second reviewer to ensure the consistency and accuracy of the extracted data. Any discrepancies were resolved through discussion and, where necessary, through consultation with a third reviewer.

### General data charting

This review compiled general data on TAR-related descriptive and economic information from various studies conducted in laboratory, clinical, and home settings. Data included details about the authors, study design (randomised controlled trial, quasi-experiment, cohort, case report, case series), sample type (stroke survivors, able-bodied individuals), and sample size. Information was also summarised on the type of TAR (actuated and non-actuated) and its target limb (upper, lower, or both). Furthermore, we examined TAR-related details, including the total duration and dose (weeks, sessions, minutes), therapist-to-patient ratio (T/P) per TAR session (ranging from 0 to 1). When applicable, we looked into the duration of the complementary CT in a clinic for participants in the TAR group. Based on whether TAR was paired with a complementary CT in a clinic, we categorised it as either standalone or adjunct TAR.

Simultaneously, we extracted a brief description of the comparator group. In clinical settings, most of the included studies had a comparator group receiving standard of care (i.e., face-to-face CT in clinic) delivered by trained therapists. Therefore, to better understand the reduction in the healthcare workforce needed for TAR versus the standard of care, we introduce a metric to calculate the relative Therapist-to-Patient (rT/P) ratio, as shown in Eq. (1). If TAR was used in combination with CT, we also accounted for the additional CT session time that the TAR group received.

1 $$\begin{aligned}\:rT/P&=({T/P}_{\_in\_TAR}*{Session\_Time}_{\_of\_TAR}\cr&\quad+{T/P}_{\_CT} *{added\_Session\_Time}_{\_of\_CT})\cr&\quad\:/\left({T/P}_{\_in\_CT}*{Session\_Time}_{\_of\_CT}\right)\end{aligned}$$

Where,

- *T/P_*_*in*_*_*_*TAR*_ or *T/P_*_*in*_*_*_*CT*_ represents the therapist-to-patient ratio during the respective TAR or CT session.
- *Session_Time_*_*of_TAR*_ or *Session_Time_*_*of_CT*_ is the total minutes respectively per TAR or CT session, when the therapist attends for stroke survivors.
- *added_Session_Time*_*_of_CT*_ is considered when TAR was used in combination with CT. It describes the additional total minutes of CT training during which therapists attend to stroke survivors in the TAR intervention group.

### Synthesis of data on TAR devices’ economic cost

The data on economic cost were synthesised descriptively for the TAR devices. Reported costs were categorised based on research prototypes and commercially available devices used in the studies. For the research prototypes, extracted values were classified as production costs, whereas the original study described costs related to hardware components, materials, assembly, and software. For commercially available TAR devices, extracted values were classified as retail prices when the original studies reported the market price, listed price, or acquisition cost of the device. These prices may include the cost of regulatory approvals, large-scale manufacturing costs, warranty and support, user training, and profit margins. Therefore, production costs and retail prices were treated as distinct cost structures and interpreted descriptively rather than as directly comparable estimates. Further, we looked into data on device cost breakdown analysis. A cost breakdown analysis provides details on each cost component mentioned earlier.

Both the production cost and retail price of the TAR devices were summarised as a median (Mdn) and range of costs by type (actuated and non-actuated) of TAR for each setting. Cost estimates in currencies other than US$ were converted using an average exchange rate of the respective year (cost data or clinical data collection date) from the Federal Reserve System of the United States of America [20].

### Quality assessment of TAR-related health-economic analyses

We assessed the reporting quality of the included health-economic analyses by checking their compliance with the Consolidated Health Economic Evaluation Reporting Standards 2022 (CHEERS 2022), a 28-item checklist [21]. The studies were classified based on compliance with the CHEERs checklist: (1) Low quality, if reported items were less than 15 items (< 50%), (2) Moderate, if reported items were 15 to 19 items (50% -70%), (3) High, if reported items were equal or more than 20 ( > = 71%) out of 28-items [22, 23]. Also, reporting quality was evaluated according to the clustered items: title (1st item), abstract (2nd item), introduction (3rd item), methods (from 4th to 21st items), results (from 22nd to 25th items), discussion (26th item), funding (27th item) and conflict of interests (28th item).

### Descriptive data on TAR-related health-economic analyses

The reported health-economic analyses were further investigated in depth. We compiled data about the type of reported health-economic analyses. There are various types of health-economic analysis, depending on the type and number of outcomes considered in the analysis [24]. The analyses are classified into five categories: (1) Cost-minimisation: If both the TAR and CT groups have or are assumed to have similar health outcomes, then the analysis compares the costs between standalone or adjunct TAR and standard of care – only CT in a clinic. (2) Cost-consequence: the analysis aims to determine whether TAR, relative to CT, reduces cost and improves multiple health outcome measures. (3) Cost-effectiveness: the study aims to establish TAR as a cheaper and more effective alternative to CT. The effectiveness of the interventions is measured in a single natural unit (i.e., life years gained). (4) Cost-utility: this subtype of cost-effectiveness analysis compares TAR to CT based on cost and health outcomes. The health outcomes are reported using a generic health outcome measure, taking into account the impact on both morbidity and mortality (i.e., quality-adjusted life years gained). (5) Cost-benefit analysis: this compares TAR’s cost and effectiveness to CT. Both cost and effectiveness outcomes are measured in monetary values (i.e., stroke survivors’ willingness to pay per gained quality adjusted healthy life year) [24]. Furthermore, we examined the studies that further analyse the budget impact of TAR implementation on health systems [25, 26].

### Synthesis of data on TAR-related health-economic analyses

Finally, we summarised data from the included health-economic analyses on outcomes per type of TAR compared to the standard of care (CT in clinic).

#### TAR-related health-economic analyses in clinical settings

In clinic settings, the costs per session for both TAR and CT were summarised as Mdn (range). As costs varied across countries and health system settings, we tried to systematise cost outcomes. We looked into cost-savings (%) associated with TAR in a clinic, as shown in formula (2) - the incremental difference between the cost of standard of care sessions and standalone TAR sessions. Positive cost-savings (%) values indicate that TAR in a clinic is a cheaper option than the comparator, while negative cost-savings (%) values indicate that TAR is more expensive than the comparator.

2 $$\begin{aligned}\:{Cost-savings}_{\_in\_clinic}\left(\%\right)&=({Avr\_Cost}_{\_of\_CT\_session}\cr&\quad-{Avr\_Cost}_{\_of\_TAR\_session})\cr&\quad/{Avr\_Cost}_{\_of\_CT\_session}\end{aligned}$$

Where,

- *Avr_Cost_*_*of*_*_*_*TAR_session*_ is the per-session average cost of standalone TAR in the clinic.

If the cost per TAR session in the clinic was not reported, the total cost of the TAR therapy was divided by the number of sessions performed in the clinic.

- *Avr_Cost*_*_of_CT_session*_ is the per-session average cost of CT in the clinic.

When the cost per standard of care session was omitted or nullified, i.e., because both the adjunct TAR and comparison groups received the same standard of care treatment, we estimated the per-session cost of standard of care based on the reported hourly cost of the workforce involved.

Since most studies investigated health-economic benefits of TAR in the clinical setting, we used these data to estimate a Spearman’s rank correlation coefficient (rho) to assess the relationship between TAR-related (i) cost savings and (ii) reduction in workforce demand, as proxied by rT/P. In a separate Spearman correlation analysis, we further investigated whether TAR-related cost savings were associated with the cost of a standard of care session. A p-value < 0.05 was considered statistically significant.

#### TAR-related health-economic analyses in home settings

Similarly to clinical settings, in home settings, the average total costs were summarised as the Mdn (range). Cost-savings (%) with TAR in home were estimated using formula (3) as an incremental difference between the total cost of TAR and its comparator. Positive cost-savings (%) values indicate that TAR is cheaper than the comparator, while negative values present TAR as more expensive than the comparator.

3 $$\begin{aligned}\:{Cost-savings}_{\_in\_home}\left(\%\right)&=({Avr\_Total\_Cost}_{\_of\_comparator}\cr&\quad -{Avr\_Total\_Cost}_{\_of\_TAR\_home})\cr&\quad/{Avr\_Total\_Cost}_{\_comparator}\end{aligned}$$

Where,

- *Avr_Total_Cost_*_*of*_*_*_*TAR_home*_ is the average total cost of TAR in home.
- *Avr_Total_Cost*_*_of_comparator*_ is the average total cost of the comparator (CT in the clinic or self-rehabilitation in home or in a clinic).

When applicable, the differences between cost components (i.e., workforce, transportation, health and social service use) were reported separately to distinguish TAR’s and CT’s impacts on each component.

## Results

### Study selection

**Fig. 1:**
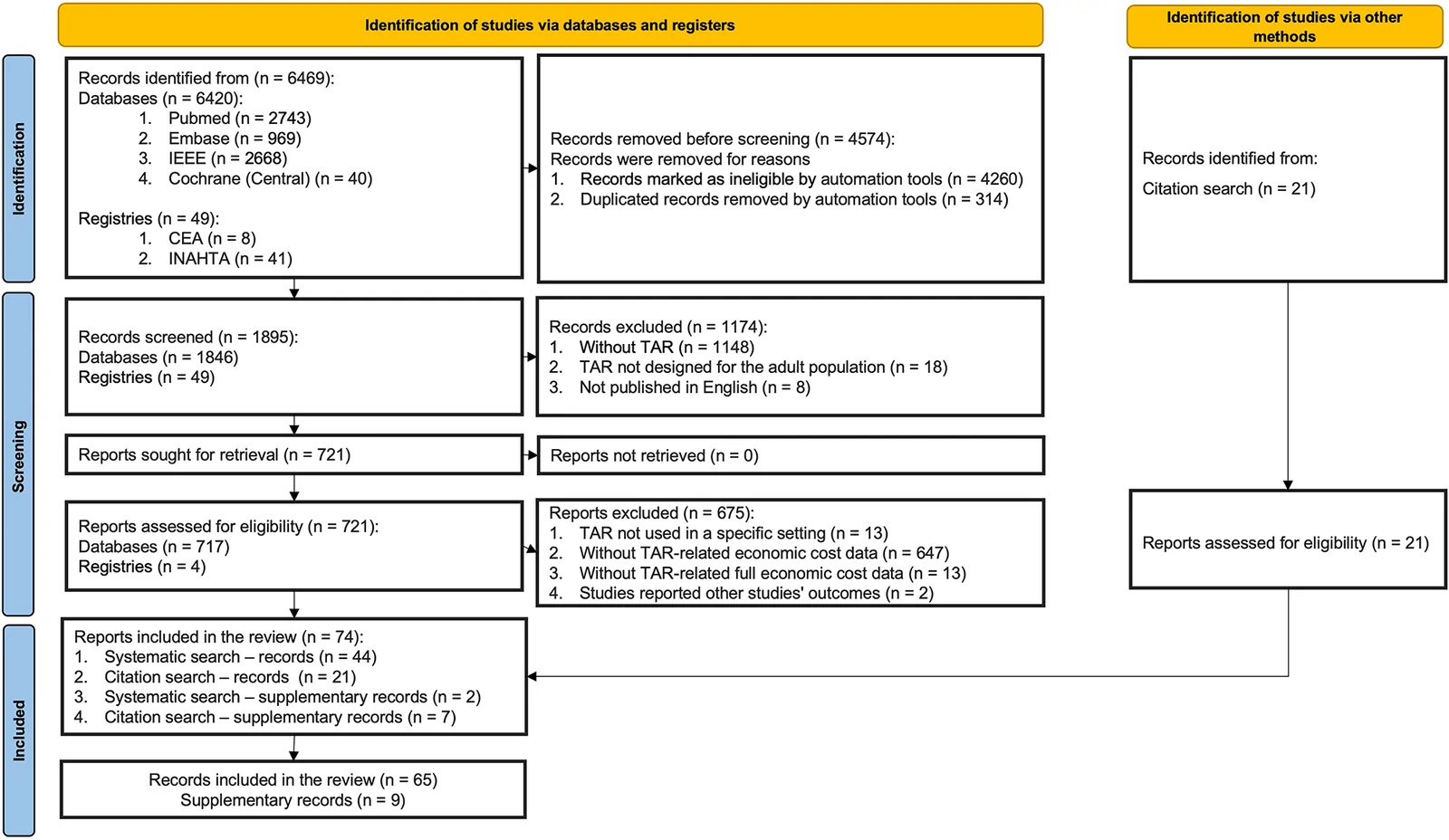
Modified PRISMA flowchart of the screening process

The screening process was charted according to the PRISMA flow diagram [27] (Fig. 1). The systematic search identified 6469 records. Before screening, 4260 records were removed as ineligible by automated filters embedded in the databases and registries, and 314 were marked as duplicates. The remaining 1895 identified records were screened. For the 10% independently screened subset, reviewer agreement was 95.8% across 190 records, and Cohen’s kappa (κ = 0.86) indicated strong inter-reviewer agreement. Disagreements were resolved through discussion. Of all screened records, 721 were sought for retrieval. After a full-text review, 675 records were excluded because they did not examine TAR or lacked cost-related data. Together with 21 eligible records identified through citation searching, we included a total of 65 records focusing on TAR-related economic costs or cost analysis (Fig. 1).

Of these, 63 records described a single study performed in a specific setting [28–90], and two reported TAR in two different settings. Khoo et al. tested the technology in the laboratory and clinical setting [91]. Also, Ates et al. used technology in the laboratory and deployed it in the home setting [92]. We considered the studies conducted in two different settings as two studies. Thus, the final data extraction and synthesis included 67 studies.

Notably, nine additional records [93–101] (Fig. 1) were used to supplement extracted information on health and economic outcomes from the six included studies. The detailed health outcomes and part of the economic outcomes of Fernandez-Garcia et al. [69] were reported by Rodgers et al. [93, 94] and extracted from there; a part of the economic outcomes of Lo et al. [70] was reported by Wagner et al. [95], and some details on the health outcomes of Islam et al. [77] were reported in Brunner et al. [98]. Similarly, for Masiero et al. [74], details were described in previous studies by Masiero et al. [96, 97]. Aguirre-Ollinger et al. [88] derived partial data on health outcomes from Budhota et al. [101], and Smayda et al. [86] from Awad et al. [100] and Smayda et al. [99].

A subset of 15 of the 65 included records (23%) was independently extracted by the second reviewer to ensure the consistency and accuracy of the extracted data. The reviewers agreed on the extraction of 13 of the 15 records (86.7%); discrepancies in the remaining records were resolved through discussion.

In a laboratory setting, 34% of the included studies (13 out of a total of 38 studies) used actuated upper limb TAR [48, 50–61], either stand-alone exoskeletons (*n* = 5) or combined with different systems (*n* = 5), and end-effector robots (*n* = 3) (Table 1). Also, the majority (10 out of 16 studies) in the clinical setting used actuated upper limb TAR [65–74], i.e., exoskeletons (*n* = 3) and single- (*n* = 4) or multiple-end-effector robots (*n* = 3) (Table 1). In contrast, the studies in the home setting used mostly (9 out of 13 studies) non-actuated TAR [79–86, 92], i.e., VR, VR combined with sensor systems or only sensor systems, most frequently for the upper limb (*n* = 6) [79, 81–84, 92] (Table 1).

### TAR devices’ production cost

The economic cost of TAR devices was extracted as a production cost or a retail price from most (63 out of 67) of the included studies (Table 1). These two cost categories were treated as different cost constructs and were synthesised descriptively, but it is important to note that they cannot be directly compared.

**Fig. 2:**
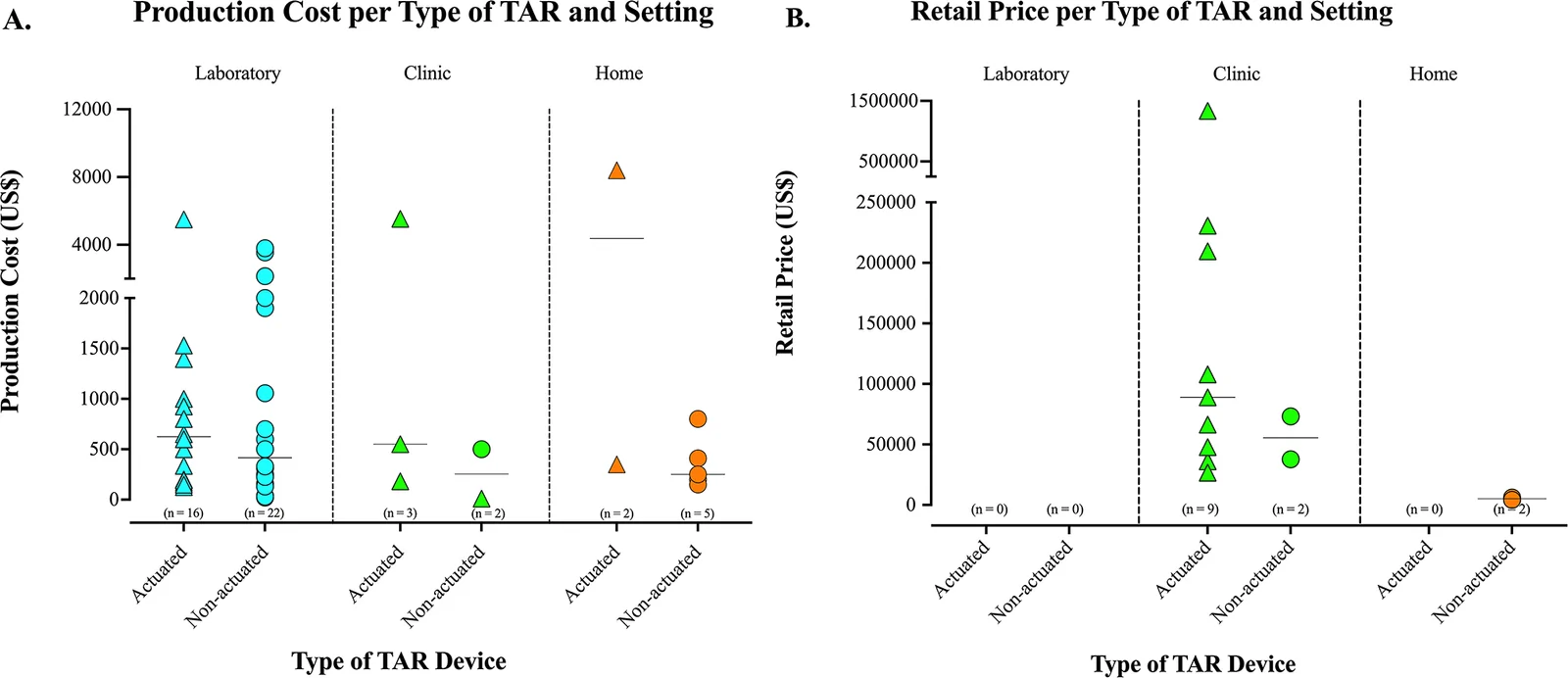
Production cost (**A**) and retail price (**B**) are shown for actuated vs. non-actuated Technology-Assisted Rehabilitation (TAR) devices that were tested in the laboratory, clinical or home settings. Fig. **A** shows that, in the laboratory and clinical settings, the median (Mdn) production costs (depicted as a horizontal line per column) for actuated and non-actuated prototypes were comparable. However, in the home settings, the cost of an actuated device was relatively higher than the Mdn cost of non-actuated ones. A similar pattern is seen in Fig. **B** for the Mdn retail price of actuated and non-actuated TAR devices in the clinical settings. In the home settings, the retail price was only reported in two studies. No studies used commercial analogues in laboratory settings

All studies in the laboratory setting and nearly half (7 out of 13) studies in the home setting used prototypes of TAR devices and reported production costs, which summarise hardware, assembly, and software costs (Fig. 2A; Table 1).

In contrast, the majority (11 out of 16) studies in the clinical setting and half (6 out of 13) studies in the home setting used commercially available TAR devices and most of them reported retail price, a combination of cost of regulatory approvals, large-scale manufacturing, warranty and support, user training, and profit margins (Fig. 2B; Table 1).

Production costs differed substantially between studies, but there were no apparent systematic differences between actuated and non-actuated prototypes or devices used in laboratory, clinical, or home settings. From the complete data in the laboratory setting, we found that the Mdn production cost of TAR prototypes was comparable between actuated (Mdn = US$ 625) and non-actuated devices (Mdn = US$ 415) (Fig. 2A; Table 1). Only five studies reported production costs in the clinical setting (Table 1; Fig. 2A). Of these, the cost of the end-effector robot (US$ 5534) was ten times higher than that of the actuated exoskeletons (Mdn = US$ 366) (details in Table 1). Also, in the home setting, production costs were low for all non-actuated (5 with production costs) TAR prototypes (Mdn = US$ 250) (Fig. 2A; Table 1). An exception was the study of Sivan et al. [89], which reported substantially higher production costs (US$ 8400) for an end-effector robot for the upper limb.

### TAR devices’ retail price

The retail prices were mainly reported for studies in the clinical settings (Fig. 2B; Tables 1 and 2, Appendix Table 3), mostly for actuated devices targeting only the upper limb (11 out of 16 studies). The retail price differed substantially across studies, with the highest amount of US$ 1336300 being reported for a complex robotic gym consisting of two end-effector devices. Mdn retail price for actuated devices across all (*n* = 9) studies was US$ 89000, which is still 100 times higher than the reported Mdn US$ 550 production costs for actuated (*n* = 3) devices (Fig. 2A-B). In the home setting, the retail price of non-actuated (*n* = 2) TAR devices was Mdn US$ 5179, whereas retail prices for actuated devices were not reported. Those without retail prices provided rental fees for non-actuated TAR devices, ranging from US$175 to US$1450 for 5–6 weeks [82, 86]. Rental fees for actuated TAR devices (1 study) fell within the range (an average of US$ 505 for 3 months) [87] (Table 1, Appendix Table 4).

**Table 2:** Health-economic analyses in clinical settings

Authors, Year	Cost-analysis’ type	Intervention						Comparator	Economic Outcomes			Health Outcomes		
		(i) Standalone TAR or (ii) Adjunct TAR to CT			Added CT in a Clinic to TAR	T/*P* of TAR	Relative T/*P* of Intervention	CT in a Clinic	CT Cost per Session	TAR Cost per Session	Difference in Cost per Session TAR vs. CT	(i) Standalone TAR or (ii) Adjunct TAR to CT Compared to CT in a clinic		
		*N* Weeks	*N* Total Sessions	Min/Session	Min/Session			Min/Session	US$	US$	%		Post Intervention	Follow-up
									**(a)**	**(b)**	**((a-b)/a)**			
Bustamante Valles K. et al., 2016 [64]	CM	6–8 *(i)*	24	120	0	0.25	0.25	120	19.2	7	64	1.U *(i)* TAR vs. CT in clinic 2. L *(i)* TAR vs. CT in clinic	1.non-inferior *at 8 weeks* 2. superior *at 8 weeks*	NA
Hesse S. et al., 2014 [65]	CM	4 *(ii)*	20	30	30	0.29 *(Estimated)*	0.65	60	13.3*	5.5* *(Standalone TAR)*	59	U *(ii)* TAR vs. CT in clinic	non-inferior *at 4 weeks*	non-inferior *at 3 months*
Pila O. et al., 2022 [66]	CM *(assumed)*	6 *(ii)*	31	60	60	0.50	0.75	120	81.2* *(Estimated)*	82* *(Estimated)*	-1	NA	NA	NA
McCabe J. et al., 2015 [67]	CM	12 *(ii)*	60	90	210	0.33	0.8	300	76.2 *(Estimated)*	94.8 *(Estimated)*	-24	U *(ii)* TAR vs. CT in clinic	non-inferior *at 12 weeks*	NA
Vanoglio F. et al., 2017 [68]	CM	6 (*i)*	30	40	0	0.3	0.3	40	17.8* *(Estimated)*	8.8* *(Estimated)*	51	U (*i)* TAR vs. CT in clinic	superior *at 6 weeks*	NA
Fernandez-Garcia C. et al., 2021 [69, 93, 94]	CU	12 *(ii)*	36	45	45	1	1	90 *(CT- enhanced therapy group)*	51.9* *(Estimated)*	106.6* *(Estimated)*	-105	U *(ii)* TAR vs. CT in clinic	non-inferior *at 12 weeks*	non-inferior *at 6*,* 12 months*
Lo A.C. et al. 2010 [70, 95]	CU	12 *(i)*	36	60	0	0.25	0.25	60 *(CT - intensive comparison therapy group)*	218.0	140.0	36	U (*i)* TAR vs. CT in clinic	non-inferior *at 12 weeks*	non-inferior *at 6*,* 9 months*
Masiero S. et al. 2014 [74, 96, 97]	CM	5 *(ii)*	25	40	80	0.5 *(Estimated)*	0.84	120	24.9*	21.4* *(Estimated)*	14	U *(ii)* TAR vs. CT in clinic	non-inferior *at 5 weeks*	non-inferior *at 3* [96, 97] *and 7 months* [96]
Shankar R. et al., 2025 [75]	CU	2 (ii)/ 24 (i) used in CU	12 (+ 6) / 7 (i) used in CU	30	15–30	1	1	45–60 (CT)	48.7 *(Estimated)*	109 *(Estimated*,* Standalone TAR)*	-124	L (ii) TAR vs. CT in clinic	non-inferior at 12 weeks	*NA*
Islam M. K. et al., 2019 [77, 98]	CM	4 *(ii)*	15	60	120	0.25	0.75	180	36.7 *(Estimated)*	34.1 *(Estimated)*	7	U *(ii)* TAR vs. CT in clinic	non-inferior *at 4 weeks* [98]	non-inferior *at 3 months* [98]
Rémy-Néris O. et al., 2021 [78]	CU	4 *(ii)*	20	60	90	0.1 *(Estimated)*	1.07	60 (SR), 90 (CT)	40.0 *(Estimated)*	94.6* *(Estimated)*	-136.5	U (*ii)* TAR vs. SR + CT in clinic	non-inferior *at 4 weeks*	non-inferior *at 12 months*

Table 2: Details are in Appendix Table 3

*Costs were converted from the original currency to US$

^a^ CM=Cost-Minimisation, CU=Cost-Utility, CT=Conventional therapy, L=Lower Limb, Min=Minutes, N=Number, NA=Not Applicable, NR=Not Reported, rT/P= relative Therapist-to-Patient ratio, TAR= Stroke Technology Assisted Rehabilitation, T/P= Therapist-to-Patient ratio, U=Upper limb

**Table 3:** Health-economic analyses in home settings

Authors, Year	Cost-Analysis’ Type	Intervention					Comparator			Economic outcomes					Health outcomes		
									Total Cost of Comparator	Total Cost of Intervention	Difference in Total Healthcare Cost	Transportation Cost of Comparator	Transportation Cost ofIntervention	Difference in Transportation Cost	(i) Standalone TAR or (ii) Adjunct TAR Compared to CT in a Clinic/Other		
		TAR					CT/Other		CT/Other	TAR	TAR vs CT/Other	CT/Other	TAR				
		(i) Standalone TAR or (ii) Adjunct TAR to CT			Added CT in a Clinic	Total Therapist Time in TAR										Post Intervention	Follow-up
		NWeeks	N Sessions	Min/Session	Min/Session	Total Min	N sessions	Min/Session	(US$)	(US$)	(%)	(US$)	(US$)	(%)			
									(a)	(b)	(a-b)/a	(c)	(d)	(c-d)/d			
Veras M. et al., 2020 [81]	NA	4*(i)*	20	30	-	75	20	30*(SR)*	NR	376.7*(Estimated)	NR	NR	26.5*(Estimated)	-	U *(i)* TAR vs SR at home	NR	NR
Lloréns R. et al., 2015 [80]	CM	7*(ii)*	20	45	NR	98	20	45*(CT in clinic)*&Usual therapy*(amount NR)*	1490.2	835.6	44	1308	0	100	L *(ii) *TAR vs CT in clinic	non-inferior at *8 weeks*	non-inferior at *3 months*
Adie K. et al., 2017 [82]	CU	6*(ii)*	30	45	NR*(Usual therapy)*	-	30	45(SR)&CT*(amount NR)*	0	175.3*	NA	-	-	-	U *(ii)* TAR vs SR at home + CT in clinic	non-inferior at *6 weeks*	non-inferior at *6 months*
Smayda K.E. et al., 2024 [86, 99, 100]**, ***	BIA**	8***(i)	24***	30***	NR	NR	24	30*(CT in clinic)*	1396.3	2320	-66	-	-	-	L *(i) *TAR vs CT in clinic	Assumed superior at *5 weeks* [100]***	NA
Housley S.N. et al., 2016 [87]	CM	12*(i)*	31	60-120	NR	NR	*36-39* *(assumed)*	60 *(assumed CT in clinic)*	1563.4	761.2	51.3	2056.7	506.9	75	NA	NA	NA
Aguirre-Ollinger G. et al., 2024 [88, 101]	CE & BIA	5 *(ii)*	30	60-120	60(in clinic)	550-640*(remote monitoring 100 min)*	28*(assumed)*	60 min* (CT in clinic) *& 60*(Usual therapy)*	2314.5*	1753*	24.3	264.7*	66.8*	75	U *(ii) *TAR vs CT in clinic	non-inferior at *5 weeks*	non-inferior at *6 months*
Fusari G. et al., 2025 [79]	CU	12	NA	NA	NR (Usual therapy)	*NR*	*NR (Usual therapy)*	*NR (Usual therapy)*	0	2017.1	NA	-	-	-	U (ii) TAR vs SR at home	non-inferior at 12 weeks	-

Table 3: Details are in Appendix Table 4

*Cost were converted from the original currency to US$. All numbers are rounded off to one decimal

**Cost estimates derived from BIA

*** TAR at home was not evaluated. However, health outcomes related to TAR at home were assumed to be similar to those of TAR in a clinic and superior to CT in a clinic, as shown by Awad et al., when comparing CT in a clinic to fully supervised TAR in a clinic [82]

^a^ BIA=Budget Impact Analysis, CE=Cost-Effectiveness, CM=Cost-Minimisation, CT=Conventional Therapy, CU=Cost-Utility, L=Lower Limb, Min=Minutes, N=Number, NA=Not Applicable, NR=Not Reported, SR=Self Rehabilitation, TAR=Stroke Technology Assisted Rehabilitation, U=Upper limb

Numerous studies (*n* = 12) have documented heterogeneous data concerning maintenance and overhead costs associated with commercial TAR devices, with percentages ranging from 6% to 83% of the retail price (Appendix Tables 3 and 4). These estimates occasionally incorporate facility overhead and financing rates. The calculations were derived from differing costing assumptions and device lifespans.

One important difference between studies conducted in clinical settings versus other settings was the frequent use of the clinical “RehabGym” concept, i.e., offering training with several different technologies, which led to the high cost of the TAR [64, 65, 69, 70]. On the other hand, the concept had the advantage of workforce reduction, as one therapist could supervise multiple stroke survivors [64, 65, 70].

### TAR devices cost breakdown analyses

The reported TAR device’s cost breakdown analysis, which details the cost of hardware, assembly, software and added profit margin, differed across settings. Although most studies included device cost, either as a production cost or as a retail price, only one-third (22 out of 67; actuated *n* = 9 and non-actuated *n* = 13) have reported a cost breakdown analysis. The studies presented hardware cost as a primary driver of the prototypes’ production cost, for both types - actuated (57–100% of reported production cost) [48, 50–52, 57–60, 71] and non-actuated (94–100% of reported production cost) [29–36, 45, 46, 80, 81, 85] devices. The assembly cost, disclosed only for actuated TAR devices, accounted for 40%–43% of the reported production cost and was the second-highest cost contributor [50, 52]. In contrast, the software cost was deemed insignificant relative to the total cost of both actuated [51, 52, 57] and non-actuated [29, 32, 81] TAR devices and was frequently not reported.

One-third (23 out of 67) of the included studies [31–34, 39, 41–47, 49–51, 58–63, 84, 89], primarily (*n* = 20) in laboratory settings [31–34, 39, 41–47, 49–51, 58–62], compared the production cost of TAR prototypes with the price of commercial analogues to establish novel devices as cheaper alternatives. However, those studies could not be considered as standard health-economic analyses.

### TAR-related health-economic analyses

The number of health economic analyses on TAR varied between settings. Only a small subset (19 out of 67 studies) reported outcomes of a health-economic analysis. Most analyses were reported for clinical settings (*n* = 11) [64–70, 74, 75, 77, 78], followed by studies in the home (*n* = 7) [79–82, 86, 88, 87] (Tables 2 and 3; Appendix Tables 3 and 4).

Only one study in the laboratory setting reported a health-economic analysis [38]. However, we discarded this study from further analyses since study-relevant information was not reported (61%, according to the CHEERs 2022 checklist, Appendix Table 2), the authors did not use a standard method of health-economic analysis, and several comparisons were ill-matched.

Overall reporting quality of health-economic outcomes in clinical settings was moderate (Mdn = 68% of the CHEERS 2022 checklist items) (Appendix Table 2). A gap was observed in reported studies; a high number did not state that they performed an economic analysis (item 1 reported by 64% of studies). Also, the methodology part of the studies lacked clarity regarding the perspective of analysis (item 8 reported by 55%), discounting of outcomes (item 10–27%), reporting dates of estimated resources (item 15–55%), subgroup analysis (item 18–46% and item 19–18%) and uncertainties of the estimates (item 20–46% and item 24–27%) and engagement with stakeholders (items 21, 25–27%).

In the clinical settings, nine studies investigated actuated TAR in 1196 stroke survivors across studies [64–70, 74, 75] (Table 1). The majority (6 out of 9) of the studies compared adjunct actuated TAR to time-matched standard of care (for details, see Table 2 and Appendix Table 3). Only two studies reported outcomes of non-actuated adjunct TAR for 335 stroke survivors.

The Mdn (range) duration of the actuated TAR in clinical settings was 6 (2–12) weeks, consisting of 30 (12–60) sessions. Each session lasted 45 (30–120) minutes. In contrast, non-actuated TAR in clinical therapy was provided for 4 (4–4) weeks, with a total of 18 (15–20) sessions, each lasting 60 (60–60) minutes (Table 2).

The studies in the clinical settings employed different health-economic analyses. Seven out of 11 studies focused on cost-minimisation [64–68, 74, 77], while the other four reported cost-utility analysis outcomes [69, 70, 75, 78] (Table 2, Appendix Table 3).

Most (10 out of 11) studies in clinical settings measured the effectiveness of TAR immediately post-intervention or up to one year afterwards (Details in Table 2). Among actuated upper-limb TAR studies in a clinic, the majority (6 out of 8) reported non-inferior outcomes compared to the standard of care; while one even found TAR superior to CT [68]. Only Pila et al. [66] assumed non-inferiority of the upper-limb-actuated TAR based on the literature. Similarly to actuated TAR, non-actuated TAR in a clinic was non-inferior to the standard of care (Table 2).

To synthesise cost outcomes, we calculated cost savings for each session of standalone or adjunct TAR vs. standard of care, which varied across studies from − 136.5% (indicating higher costs for non-actuated TAR vs. standard of care) to 64% (indicating that TAR is cost-saving relative to standard of care) (Table 2, Appendix Table 3).

**Fig. 3:**
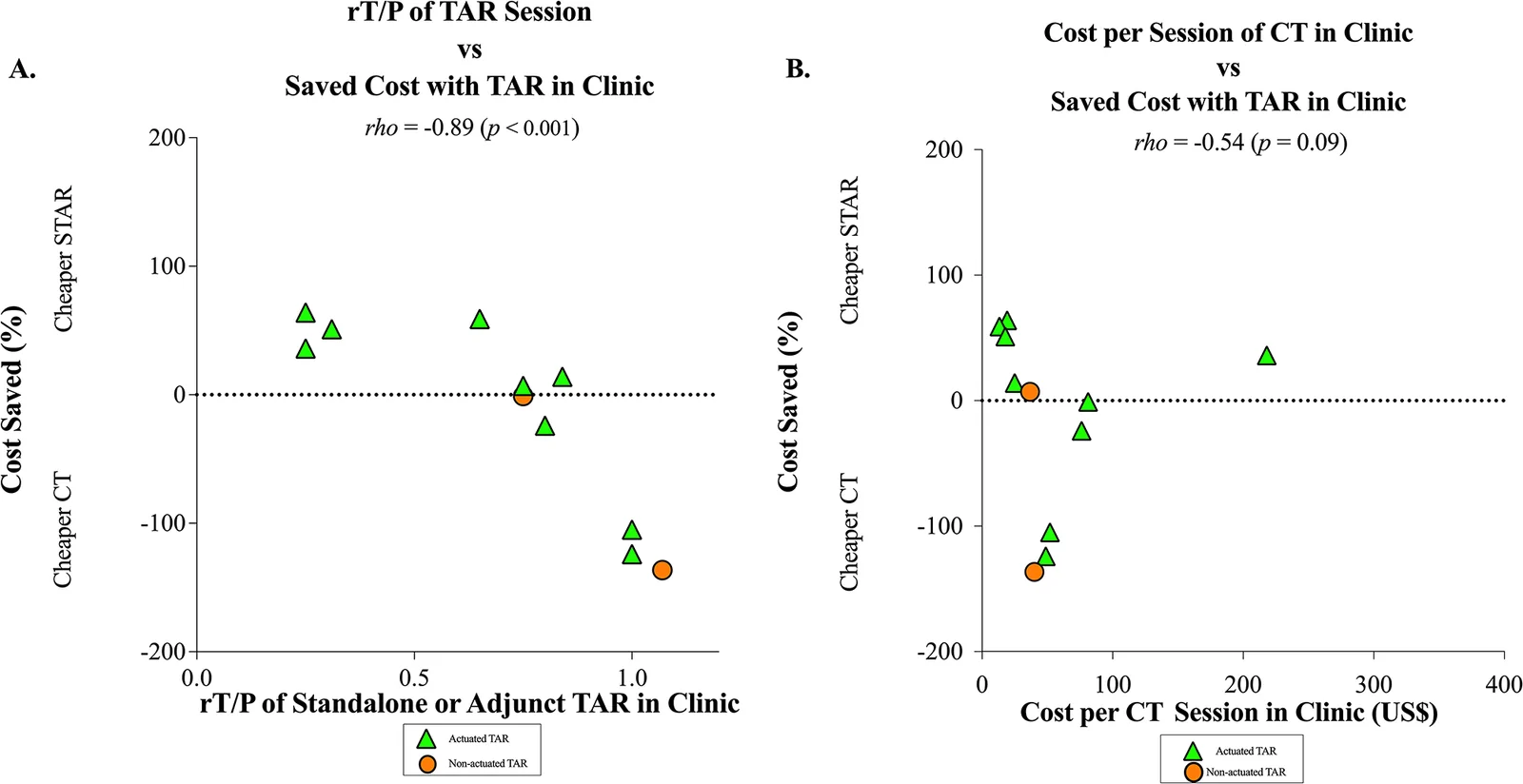
Fig. **A** shows that in the clinical settings, the application of Technology-Assisted Rehabilitation (TAR) resulted in reduced manpower, which is reflected in a lower relative Therapist-To-Patient Ratio (rT/P). A lower rT/P also correlated with a lower TAR cost (Spearman’s correlation (rho)=-0.89, *p* < 0.001). However, as Fig. **B** shows the cost of Conventional Therapy (CT) in clinic did not significantly influence the overall cost reduction linked to TAR in clinic (Spearman’s correlation (rho)=-0.54, *p* = 0.09)

We further explored whether a reduction in workforce demand, with standalone or adjunct TAR, could explain this considerable variation in cost savings across studies (Fig. 3A; Table 2, Appendix Table 3). We expressed the T/P for standalone TAR or adjunct TAR to CT in the clinic relative to the T/P for the standard of care. A rT/*P* < 1 indicates that TAR reduces the demand for the workforce. In contrast, a rT/P > = 1 indicates the same or higher workforce is needed for TAR compared to the standard of care (see methods for details). The latter was observed when the actuated TAR session was delivered in a fully therapist-supervised mode [69, 75] (T/*P* = 1) or when the standard of care–CT in clinic included an unsupervised self-rehabilitation component [78] (T/*P* > 1). Figure 3A shows that TAR devices with low relative T/P are cost-saving (Spearman’s correlation (rho)=-0.89, *p* < 0.001).

The studies also varied in their estimated cost per standard of care session, which ranged from US$13.3 to US$218 (Table 2, Appendix Table 3). The effect of the absolute cost of a standard of care session on total cost savings with TAR only reached a statistical trend level (Spearman’s correlation (rho)=-0.54, *p* = 0.09).

In the home setting, seven cost analyses were reported out of all 11 included studies (Table 3). Reporting quality was high (Mdn = 75% of the CHEERS 2022 checklist items, Appendix Table 2). While most of the studies were identified as economic analysis in the title (item 1 reported by 71%), similar to clinical settings, a gap was observed in the methodology, which lacked clarity regarding the discounting of outcomes (item 10–14%), subgroup analysis (item 18–14%), addressing uncertainties of the estimates (item 20 and 24 only at 29%) and reporting outcomes of engagement with stakeholders (items 25–57%) and disclosing conflict of interest (item 28–71%).

In home settings, included health-economic studies reported outcomes of 449 stroke survivors using non-actuated TAR devices (Tables 1 and 3, Appendix Table 4) [79–82, 86]. Mdn (range) of 22 (20–30) non-actuated TAR sessions in home settings were prescribed for 7 (4–12) weeks. A session’s duration was 38 (30–45) minutes. In contrast, studies reporting outcomes of an actuated TAR therapy in home settings measured among 73 stroke survivors after 9 (5–12) weeks of TAR, consisting of a total of 31 sessions, each lasting 60–120 min (Table 3, Appendix Table 4).

Overall, reported TAR use in home settings varied between 1020 and 2763 min or 29.3%–75.5% usage rate of the total study period [82, 87, 88] (Appendix Table 4). All studies implemented unsupervised remote TAR. During the TAR interventions, the therapists assessed the stroke survivors’ performance remotely. In this activity, the therapists spent a total of 75–100 min [80, 81, 88] (Table 3, Appendix Table 4). Few studies provided additional CT in a clinic for the TAR group [79, 82, 88].

Comparators to TAR varied across home-setting studies. Most (5 out of 7) studies used [79, 80, 88] or assumed standard of care (CT in a clinic) [86, 87], while others used self-rehabilitation [81, 82]. The type of cost analysis varied: while two of the studies focused on cost minimisation [80, 87], few of them performed or modelled cost-effectiveness analysis combined with budget impact analysis (BIA) [86, 88], and another two reported cost-utility outcomes [79, 82] (Table 3, Appendix Table 4).

Similar to the clinical settings, about half (4 out of 7) of the studies in the home setting showed that health-economic outcomes (up to 12 months) did not differ statistically between remote, actuated or non-actuated TAR and comparators [79, 80, 82, 88] (Table 3, Appendix Table 4). However, TAR saved [80, 88] or demonstrated the potential [87] to save 24%–51% of costs, as it could reduce the need for therapists compared to CT in a clinic. Further cost reduction of 75%–100% [80, 87, 88] is linked to decreased transportation needs (Table 3, Appendix Table 4). According to BIA outcomes, the total cost for public payers could be reduced by 0.2% [86] and 5% [88] when adopting TAR in home compared to the standard of care. The cost reduction mainly depends on the uptake of the service, as there is a need for a minimum increase of patient share with TAR, respectively, by 5 [86] and 20% [88] in the first year. However, as demonstrated by few studies, other factors such as clinical outcomes, hospital readmission rates, and the duration of TAR service could influence cost reduction rates [79, 86].

## Discussion

We reviewed existing studies that reported on the economic and health outcomes associated with actuated and non-actuated devices for TAR developed in laboratories or applied in clinical and home environments.

Of the 67 studies included, only 19 reported health-economic analyses. Most information on TAR’s comparable effectiveness and cost savings relative to the standard of care was derived from clinical settings (*n* = 11 studies). Only few studies investigated TAR in home (*n* = 7). Most of these studies suggested that there is potential for cost reduction but offered limited data on relative effectiveness, limiting interpretability and relevance.

We noted that early health-economic analyses are rarely conducted in laboratory settings. Therefore, studies conducted in laboratory settings need to be interpreted separately from those conducted in clinical and home settings. In these studies, economic information was generally limited to prototype hardware component costs or assembly expenses, rather than comprehensive economic evaluations, service delivery costs, or implementation-related expenses. This focus primarily reflects an emphasis on technical validation of research prototypes, often aimed at refining hardware choices and simplifying assembly to reduce production costs compared to similar devices. Presently, estimating future cost-benefit in the intended use case and setting appears to be an afterthought. The comparison of prototype production costs with retail prices was intended to explore how device costs evolve from development through commercialisation and practical application, rather than to assess cost comparison or real-world economic benefits.

However, this narrow focus on reducing hardware costs also highlights the need to consider broader economic and implementation implications in the early development cycle. In particular, our review suggests that, where clinically appropriate, some TAR devices may offer economic advantages by supporting reduced-supervision models, including scenarios in which one therapist supervises multiple stroke survivors (see below). However, this is not a universal design objective, and not all TAR devices are currently suitable for semi- or unsupervised use. Suitability may depend on device capabilities, patient characteristics, treatment protocols, safety standards, usability, scalability, and integration into existing health systems. Performing early health-economic analysis alongside device testing in a laboratory allows for ongoing feedback between technical development and economic assessment. This integrated method aids in refining device design and capabilities based on anticipated cost outcomes which can be derived from expert opinions or simulations. It also helps to identify appropriate clinical use cases, to optimise resource allocation, to define the logistics of clinical implementation, to provide evidence-based guidance for upcoming clinical trials and deployment planning and to facilitate future technology transfer to industry [102]. However, it should be noted that early health-economic studies in the laboratory setting would benefit from harmonising evaluation frameworks, i.e., by standardising protocols and experimental designs, to improve comparability and policy relevance across studies [103].

Most reviewed studies reporting health-economic analyses for commercial TAR devices took place in clinical settings. In these studies, relatively moderate compliance was observed with the CHEERS 2022 checklist reporting standards compared to other settings. Given the variability in study designs, sample sizes, TAR duration in a clinic, supervision model, and the range of measured health-economic outcomes, these findings should be interpreted with caution. Overall, the available evidence supports the view that standalone or adjunct TAR in a clinic may achieve broadly comparable clinical effectiveness and be a cost-saving alternative to the standard of care under specific service delivery conditions, particularly when one therapist supervises multiple stroke survivors in parallel. However, this conclusion is largely based on cost-comparison/cost-minimisation analyses and limited formal cost-effectiveness evidence. Therefore, the cost-effectiveness of TAR in the clinical context remains to be established.

More specifically, most studies reported that both actuated and non-actuated upper-limb TAR led to similar clinical improvements (8 out of 11) compared with the standard of care, consistent with the previous review by Veerbeek et al. [104]. Two studies showed the superiority of the actuated (exoskeleton) upper limb [68] and lower limb (robot gym, coupled functional electrical stimulation with electromechanical motor-assisted ergometer) TAR [64]. However, these studies did not further conduct a cost-effectiveness analysis.

Based on the analysed data from the examined studies, which is in line with previous reviews [14–17], the therapist-to-patient ratio per session (r*T*/*P* < 0.5) appeared to be an important determinant of standalone or adjunct actuated and non-actuated TAR-related cost savings (Table 2, Appendix Table 3; Fig. 3A). In our analysis, the cost per TAR session in a clinic was inversely associated with supervision, suggesting that semi- and minimally supervised TAR may improve service efficiency. However, this association was based on a small number of included studies and should be interpreted with caution. Furthermore, no study reported a statistically significant difference in economic outcomes between TAR and CT at post-intervention follow-up (Appendix Table 3). Given the growing demand for post-stroke rehabilitation [105], the increased use of semi- and unsupervised TAR in a clinic presents a potential strategy to reduce costs and/or enhance efficiency; However, not all healthcare systems are prepared to integrate this approach into routine post-stroke care [106]. Therefore, future studies also need to focus on the systemic impact of incorporating semi- and unsupervised TAR services in the clinic.

A limited number (*n* = 7) of studies investigated the health-economic analyses of TAR devices in the home setting. This may reflect currently limited availability of suitable devices for home use, as well as the technological and logistical challenges associated with implementing TAR in home settings [106]. The challenges include potential device failure risk, software and hardware maintenance, patient selection, therapist, patient, and caregiver training, data transfer from user to vendor and privacy concerns, and safety risks such as falls and fractures. These factors may also affect the cost of deploying TAR in home settings; however, not all studies account for them in their analyses.

The effectiveness of TAR in home settings cannot be determined conclusively from the available evidence, as included studies used heterogeneous comparators. Comparators included or assumed CT in clinic, self-rehabilitation in clinic or at home, and usual therapy, each differing in therapist and caregiver involvement, treatment intensity, travel requirements, and associated resource use. Only single studies demonstrated non-inferior effectiveness either for actuated [88] or for non-actuated [80] or assumed superiority of non-actuated [86] TAR in home settings compared to the standard of care. Studies reported that both types of TAR in home settings, actuated [87, 88] and non-actuated [80], along with remote monitoring, have the potential to reduce costs associated with the time therapists spend with stroke survivors and increase device use frequency [80, 87, 88]. The costs could be further reduced when accounting for transportation costs [80, 87, 88]. By removing the need for stroke survivors to travel to the clinic, TAR not only simplifies access to the service but also increases the convenience of the service timing [87] (Table 3, Appendix Table 4). Further, TAR could lower the associated healthcare costs, such as decreased hospital admissions [86] (Appendix Table 4). This suggests that both types of TAR in home settings could be an alternative method for delivering rehabilitation services at reduced cost. However, because comparators vary across studies, cost outcomes need to be interpreted within each study context and compared with caution. Hence, better-powered studies are needed to systematically compare clinical effectiveness and costs relative to the standard of care, ideally using randomised clinical trial designs where feasible, or pragmatic and other robust comparative designs where randomisation or control of training intensity is challenging, capturing detailed implementation, user training, maintenance, infrastructure, technical support and safety outcomes.

### Recommendation for future TAR-related health-economic analysis

One main goal of TAR is to improve health outcomes either by increasing training effectiveness or by reducing cost and/or the demand on the healthcare workforce, while making post-stroke rehabilitation more accessible [10, 49]. To effectively integrate TAR into the health system, future studies should go beyond reporting production costs, retail prices, or rental fees and conduct more in-depth cost-effectiveness analyses. These evaluations also need to incorporate long-term, real-world evidence-informed operational costs, including technical support (i.e., software and infrastructure maintenance, component replacement, consumables) and user training and support. Furthermore, broader sustainability outcomes (i.e., carbon emissions) should be considered when comparing TAR to the standard of care [24], which is usually conventional rehabilitation therapy. Adjunct TAR in home settings offers the potential to achieve a high overall training dose, as stroke survivors can train more frequently than in conventional outpatient settings at no additional cost to the base fare (i.e., rental fees). However, it remains to be tested whether a higher training dose translates into better cost-effectiveness. Ideally, such studies would also assess the economic implications of TAR beyond just the costs of the intervention and related transportation expenses. Specifically, analyses should also consider post-intervention social (i.e., survivors’ productivity losses due to unemployment and further loss of household income, while caregivers’ support in daily living activities) and healthcare (i.e., readmissions to inpatient care) expenses [24]. Furthermore, time-driven activity-based costing approaches could increase the precision of cost estimates [107], particularly for TAR implemented in clinical settings. In this way, accurately estimated average and range of time required for each activity of TAR could assist in improving the efficiency of healthcare and technical personnel to deliver gym concepts in a clinic. A delineated process will also assist healthcare managers in assessing TAR’s impact on workflow and guide them in optimising existing workforce capacity to enhance productivity.

In addition to obtaining experimental evidence, modelling different scenarios of TAR’s clinical use and its implementation strategies in the system can be used to estimate its impact on individuals and the health system [102]. In these scenarios, TAR can either substitute the existing mode of post-stroke rehabilitation (standalone TAR) or complement it (adjunct TAR). By running simulations, we can estimate TAR’s impact on reducing workforce demand and improving access to care for survivors, especially those in underserved or remote areas. Although these models have limitations, such as underlying assumptions and a lack of empirical, real-world evidence, they can provide valuable insights for refining the design-to-value alignment of healthcare innovations already at an early stage. Simulating different scenarios under the assumption that TAR is non-inferior to standard of care treatments can shed light on how TAR might positively affect long-term costs that matter to policymakers and payers, such as decreased hospital readmission rates and reduced need for social care [108]. This approach could also inform a budget impact analysis [26] with alternative scenario modelling to estimate the impact of TAR’s implementation cost on the healthcare financing system. This information is important for health authorities and third-party payers to reflect on the evolving demand for TAR therapy, allocate funds, and enhance service affordability for stroke survivors, i.e., by recognising TAR in home settings for reimbursement.

### Limitations

Although we conducted repeated searches at multiple time points and even updated the systematic search keywords to reduce their restrictiveness, approximately one-third of the included studies were still identified through citation searching, most of which were conducted in laboratory settings. These studies report only the production cost of TAR devices in the main text or the appendix and do not provide any cost analysis; this suggests that database searches and metadata-based retrieval have limited sensitivity for engineering-oriented TAR studies, where cost information is reported inconsistently. Also, the substantial difference between production costs and retail prices should be viewed with caution, as they reflect different cost constructs. Unlike prototype production costs, commercial retail prices also include cost of regulatory approvals, large-scale manufacturing, warranty and support, user training, and profit margins. Consequently, when technological innovation primarily aims to reduce production costs, one may carefully evaluate whether these reductions will translate into economic impact.

Further, performing a more detailed meta-analysis of TAR-related economic and health outcomes was not possible because the identified health-economic studies differed substantially in their research designs and health outcome measures. This limitation was compounded by the wide and diverse range of TAR interventions and CT comparators. TAR devices were classified into actuated and non-actuated groups to facilitate evidence mapping across a small set of studies, while CT was used as a practical benchmark for standard therapist-led rehabilitation. Nonetheless, both varied considerably across different health systems and studies. As a result, outcomes of comparisons between TAR and CT should be interpreted carefully, considering each study’s specific context.

## Conclusion

Limited evidence suggests that partial (adjunct TAR to therapist-managed CT in a clinic) or complete (standalone) TAR in a clinic may provide a non-inferior alternative to the current standard of care for post-stroke rehabilitation. TAR utilising in-clinic setups that enable minimally supervised or unsupervised therapy sessions may lower costs by allowing one therapist to oversee multiple stroke survivors at once. Nevertheless, further investigation is needed into the maintenance and logistical aspects of this approach.

Unsupervised adjunct TAR in home settings has the potential to minimise therapists’ presence, decrease commuting time to the clinic, enhance the service’s convenience and potentially save costs. However, health-economic evidence is scarce in the home setting and of limited quality. This could be addressed by better-designed, well-powered studies using an adequate comparator (standard of care).

In laboratory settings, most technology developers focus on reducing the production costs of prototypes, while the anticipated impact on health systems and options for scaling up the TAR concept remain largely unexplored. To narrow the gap, technology developers can consider conducting early health-economic analyses.

Therefore, future studies need to prioritise continuous health-economic analyses of the devices for TAR throughout the stages of development, introduction to the market and implementation in practice.

## Supplementary Information

Below is the link to the electronic supplementary material.

Supplementary Material 1

## Data Availability

All data analysed during this study are included in this published article and its appendix file.

## Abbreviations

BCI: Brain-Computer Interface
BIA: Budget Impact Analysis
CHEERS: Consolidated Health Economic Evaluation Reporting Standards
CT: Conventional Therapy
MCI: Muscle-Computer Interface
Mdn: Median
rT/P: Relative Therapist-to-patient ratio
TAR: Technology-Assisted Rehabilitation
T/P: Therapist-to-Patient ratio
VR: Virtual Reality
