# Supplementary material for: Cost and economic evidence of stroke technology-assisted rehabilitation: a scoping review
Source: BMC Health Serv Res. 2026 Aug 11;26:1564. doi: 10.1186/s12913-026-15267-7 (PMC13757240; doi:10.1186/s12913-026-15267-7)
Supplement: Supplementary file 1 [file 12913_2026_15267_MOESM1_ESM.docx]

| Appendix Table 1. Keywords Used for Search in Different Databases | | |  |  |  |  |  |
| --- | --- | --- | --- | --- | --- | --- | --- |
|  | | | Hits in 2022 | Hits in 2023 | Hits in 2025 | Hits in 2026 –  Old | Hits in 2026 – Updated |
| Pubmed | | |  |  |  |  |  |
| Old #1 | (stroke* or poststroke or apoplex* or cerebral vasc* or brain vasc* or cerebrovasc* or SAH or IS or TIA or ICH or acute stroke or prevalent stroke or brain* or cerebr* or hemispher* or intracran* or intracereb* or infratentorial or supratentorial or middle cerebr* or mcan* or anterior circulation or basilar artery or vertebral artery or ischem* or infarct* or thrombo* or emboli* or occlus* or hypoxi* or brain* or cerebr* or cerebell* or intracereb* or intracran* or parenchymal or intraparenchymal or intraventricular or intracerebral or infratentorial or supratentorial or basal gangl* or putaminal or putamen or posterior fossa or hemispher* or subarachnoid or haemorragh* or hemorragh* or hematoma or bleed* or hemiparesis or hemipleg* or paresis or paretic or paraparesis) AND (upper limb or upper-limb or upper extremity or upper-extremity or shoulder or arm or forearm or wrist or hand or finger or thumb or lower limb or lower-limb or lower extremity or lower-extremity or gait) AND (telemedicine or telemetry or telerehab* or tele-rehab* or telerehab or telehealth or tele-health or telehomecare or tele-homecare or teleconsultation or tele-consultation or telecare or tele-care or ehealth or e-health or m-health or mhealth or telecoaching or tele-coaching or telecomunication* or teleconsultation or videoconference or video-conference or videoconsultation or remote consultation or telespeech or tele-speech or telepractice or teletherap* or telerehab* or tele-rehab* or robot* or tech* or haptic* or kinectic* or virtual* or game* or exergame* or mobile* or appl* or vidoe* or remote* or distance* or distant* or home* or home based*) AND (rehab* or rehabilitation or physiotherapy or physical therapy or speech therapy or occupational therapy) AND (economic* or economic-modelling or economic-analysis or economic evaluation or economic study or cost* or cost minimisation or cost-utility or cost-consequence or cost-effectiveness or cost comparison or cost-benefit or cost-saving or cost-analysis or cost description or cost allocation or financial cost or dollar cost) | | 1322 | 1462 | 1701 | 2322 |  |
| Old #2 | #1 AND ((adaptiveclinicaltrial[Filter] OR booksdocs[Filter] OR casereports[Filter] OR clinicalstudy[Filter] OR clinicaltrial[Filter] OR clinicaltrialprotocol[Filter] OR clinicaltrialphasei[Filter] OR clinicaltrialphaseii[Filter] OR clinicaltrialphaseiii[Filter] OR clinicaltrialphaseiv[Filter] OR collectedwork[Filter] OR comment[Filter] OR comparativestudy[Filter] OR conferenceproceedings[Filter] OR consensusstatement[Filter] OR controlledclinicaltrial[Filter] OR correctedandrepublishedarticle[Filter] OR dataset[Filter] OR editorial[Filter] OR electronicsupplementarymaterials[Filter] OR equivalencetrial[Filter] OR evaluationstudy[Filter] OR evidencesynthesis[Filter] OR expressionofconcern[Filter] OR guideline[Filter] OR historicalarticle[Filter] OR interview[Filter] OR introductoryjournalarticle[Filter] OR meta-analysis[Filter] OR multicenterstudy[Filter] OR networkmetaanalysis[Filter] OR observationalstudy[Filter] OR personalnarrative[Filter] OR practiceguideline[Filter] OR pragmaticclinicaltrial[Filter] OR preprint[Filter] OR randomizedcontrolledtrial[Filter] OR researchsupportnonusgovt[Filter] OR researchsupportusgovernment[Filter] OR researchsupportusgovtnonphs[Filter] OR researchsupportusgovtphs[Filter] OR review[Filter] OR scopingreview[Filter] OR systematicreview[Filter] OR twinstudy[Filter] OR validationstudy[Filter]) AND (fft[Filter]) AND (humans[Filter]) AND (english[Filter]) AND (alladult[Filter])) | | 569 | 595 | 715 | 1018 |  |
| Updated #1 | (stroke* or poststroke or apoplex* or cerebral vasc* or brain vasc* or cerebrovasc* or SAH or IS or TIA or ICH or acute stroke or prevalent stroke or brain* or cerebr* or hemispher* or intracran* or intracereb* or infratentorial or supratentorial or middle cerebr* or mcan* or anterior circulation or basilar artery or vertebral artery or ischem* or infarct* or thrombo* or emboli* or occlus* or hypoxi* or brain* or cerebr* or cerebell* or intracereb* or intracran* or parenchymal or intraparenchymal or intraventricular or intracerebral or infratentorial or supratentorial or basal gangl* or putaminal or putamen or posterior fossa or hemispher* or subarachnoid or haemorragh* or hemorragh* or hematoma or bleed* or hemiparesis or hemipleg* or paresis or paretic or paraparesis) AND ( upper limb or upper-limb or upper extremity or upper-extremity or shoulder or arm or forearm or wrist or hand or finger or thumb or lower limb or lower-limb or lower extremity or lower-extremity or gait) AND (telemedicine or telemetry or telerehab* or tele-rehab* or telerehab or telehealth or tele-health or telehomecare or tele-homecare or teleconsultation or tele-consultation or telecare or tele-care or ehealth or e-health or m-health or mhealth or telecoaching or tele-coaching or telecomunication* or teleconsultation or videoconference or video-conference or video consultation or remote consultation or tele speech or tele-speech or telepractice or teletherap* or telerehab* or tele-rehab* or robot* or tech* or haptic* or kinectic* or virtual* or game* or exergame* or mobile* or appl* or vidoe* or remote* or distance* or distant* or home* or home based* ) AND (rehab* or rehabilitation or physiotherapy or physical therapy or speech therapy or occupational therapy ) AND (economic* or economic-modelling or economic-analysis or economic evaluation or economic study or cost* or cost minimisation or cost-utility or cost-consequence or cost-effectiveness or cost comparison or cost-benefit or cost-saving or cost saving or cost-analysis or cost description or cost allocation or financial cost or dollar cost or low-cost or low cost or lower cost or reduced cost or low price or cheap or cheaper or inexpensive or affordable or affordability or budget friendly or low resource or resource limited) | |  |  |  |  | 2743 |
| Updated # 2 | #1 AND ((adaptiveclinicaltrial[Filter] OR booksdocs[Filter] OR casereports[Filter] OR clinicalstudy[Filter] OR clinicaltrial[Filter] OR clinicaltrialprotocol[Filter] OR clinicaltrialphasei[Filter] OR clinicaltrialphaseii[Filter] OR clinicaltrialphaseiii[Filter] OR clinicaltrialphaseiv[Filter] OR collectedwork[Filter] OR comment[Filter] OR comparativestudy[Filter] OR conferenceproceedings[Filter] OR consensusstatement[Filter] OR controlledclinicaltrial[Filter] OR correctedandrepublishedarticle[Filter] OR dataset[Filter] OR editorial[Filter] OR electronicsupplementarymaterials[Filter] OR equivalencetrial[Filter] OR evaluationstudy[Filter] OR evidencesynthesis[Filter] OR expressionofconcern[Filter] OR guideline[Filter] OR historicalarticle[Filter] OR interview[Filter] OR introductoryjournalarticle[Filter] OR meta-analysis[Filter] OR multicenterstudy[Filter] OR networkmetaanalysis[Filter] OR observationalstudy[Filter] OR personalnarrative[Filter] OR practiceguideline[Filter] OR pragmaticclinicaltrial[Filter] OR preprint[Filter] OR randomizedcontrolledtrial[Filter] OR researchsupportnonusgovt[Filter] OR researchsupportusgovernment[Filter] OR researchsupportusgovtnonphs[Filter] OR researchsupportusgovtphs[Filter] OR review[Filter] OR scopingreview[Filter] OR systematicreview[Filter] OR twinstudy[Filter] OR validationstudy[Filter]) AND (fft[Filter]) AND (humans[Filter]) AND (english[Filter]) AND (alladult[Filter])) | |  |  |  |  | 1190 |
| Embase | | |  |  |  |  |  |
| Old #1 | ('cerebrovascular accident'/exp OR 'cva' OR 'accident, cerebrovascular' OR 'acute cerebrovascular lesion' OR 'acute focal cerebral vasculopathy' OR 'acute stroke' OR 'apoplectic stroke' OR 'apoplexia' OR 'apoplexy' OR 'blood flow disturbance, brain' OR 'brain accident' OR 'brain attack' OR 'brain blood flow disturbance' OR 'brain insult' OR 'brain insultus' OR 'brain vascular accident' OR 'cerebral apoplexia' OR 'cerebral insult' OR 'cerebral stroke' OR 'cerebral vascular accident' OR 'cerebral vascular insufficiency' OR 'cerebro vascular accident' OR 'cerebrovascular accident' OR 'cerebrovascular arrest' OR 'cerebrovascular failure' OR 'cerebrovascular injury' OR 'cerebrovascular insufficiency' OR 'cerebrovascular insult' OR 'cerebrum vascular accident' OR 'cryptogenic stroke' OR 'insultus cerebralis' OR 'ischaemic seizure' OR 'ischemic seizure' OR 'stroke' OR 'thrombotic stroke' OR 'stroke patient'/exp OR 'stroke patient' OR 'stroke sufferer' OR 'stroke sufferers') AND ('upper limb'/exp OR 'upper extremity' OR 'upper-limb' OR 'upper-extremity' OR 'lower limb'/exp OR 'lower extremity' OR 'lower-limb' OR 'lower-extremity') AND ('rehabilitation'/exp OR 'telerehabilitation'/exp OR 'e-rehabilitation' OR 'remote rehabilitation' OR 'tele-rehabilitation' OR 'telerehabilitation' OR 'virtual rehabilitation' OR 'rehabilitation care' OR 'neurorehabilitation'/exp OR 'neuro-rehabilitation' OR 'neurologic rehabilitation' OR 'neurological rehabilitation' OR 'neurorehabilitation' OR 'rehabilitation care'/exp OR 'home rehabilitation'/exp OR 'functional training'/exp OR 'muscle training'/exp OR 'muscle strengthening' OR 'muscle training' OR 'myofunctional therapy' OR 'training, muscle' OR 'occupational therapy'/exp OR 'ergotherapy' OR 'occupation therapy' OR 'occupational therapy' OR 'home rehabilitation'/exp/mj OR 'home rehabilitation' OR 'rehabilitation robot'/exp OR 'brain computer interface' OR 'muscle computer interface' OR 'rehabilitation robot' OR 'robot-assisted rehabilitation' OR 'robotic rehabilitation device' OR 'robot'/exp/mj) AND ('cost'/exp OR 'cost' OR 'cost allocation' OR 'cost sharing' OR 'costs and cost analysis' OR 'deductibles and coinsurance' OR 'cost minimization analysis'/exp OR 'cost benefit analysis'/exp OR 'cost utility analysis'/exp OR 'cost utility' OR 'cost utility analysis' OR 'societal cost'/exp OR 'societal cost' OR 'cost effectiveness analysis'/exp/mj OR 'cost effectiveness' OR 'cost effectiveness analysis' OR 'cost effectiveness ratio' OR 'cost efficiency analysis' OR 'cost benefit analysis'/exp/mj OR 'cost analysis' OR 'cost benefit' OR 'cost benefit analysis' OR 'cost benefit ratio' OR 'cost-benefit analysis' OR 'cost minimisation' OR 'cost minimisation analysis' OR 'cost of illness'/exp/mj OR 'cost of illness' OR 'cost of illness analysis' OR 'economic aspects of illness' OR 'cost consequence analysis'/exp/mj) | | 425 | 471 | 596 | 826 |  |
| Old #2 | #1 AND ([article]/lim OR [review]/lim) AND ([adult]/lim OR [young adult]/lim OR [middle aged]/lim OR [aged]/lim OR [very elderly]/lim) AND [english]/lim | | 137 | 146 | 164 | 206 |  |
| Updated #1 | ('cerebrovascular accident'/exp OR 'cva' OR 'accident, cerebrovascular' OR 'acute cerebrovascular lesion' OR 'acute focal cerebral vasculopathy' OR 'acute stroke' OR 'apoplectic stroke' OR 'apoplexia' OR 'apoplexy' OR 'blood flow disturbance, brain' OR 'brain accident' OR 'brain attack' OR 'brain blood flow disturbance' OR 'brain insult' OR 'brain insultus' OR 'brain vascular accident' OR 'cerebral apoplexia' OR 'cerebral insult' OR 'cerebral stroke' OR 'cerebral vascular accident' OR 'cerebral vascular insufficiency' OR 'cerebro vascular accident' OR 'cerebrovascular accident' OR 'cerebrovascular arrest' OR 'cerebrovascular failure' OR 'cerebrovascular injury' OR 'cerebrovascular insufficiency' OR 'cerebrovascular insult' OR 'cerebrum vascular accident' OR 'cryptogenic stroke' OR 'insultus cerebralis' OR 'ischaemic seizure' OR 'ischemic seizure' OR 'stroke' OR 'thrombotic stroke' OR 'stroke patient'/exp OR 'stroke patient' OR 'stroke sufferer' OR 'stroke sufferers') AND ('upper limb'/exp OR 'upper extremity' OR 'upper-limb' OR 'upper-extremity' OR 'lower limb'/exp OR 'lower extremity' OR 'lower-limb' OR 'lower-extremity') AND ('rehabilitation'/exp OR 'telerehabilitation'/exp OR 'e-rehabilitation' OR 'remote rehabilitation' OR 'tele-rehabilitation' OR 'telerehabilitation' OR 'virtual rehabilitation' OR 'rehabilitation care' OR 'neurorehabilitation'/exp OR 'neuro-rehabilitation' OR 'neurologic rehabilitation' OR 'neurological rehabilitation' OR 'neurorehabilitation' OR 'rehabilitation care'/exp OR 'home rehabilitation'/exp OR 'functional training'/exp OR 'muscle training'/exp OR 'muscle strengthening' OR 'muscle training' OR 'myofunctional therapy' OR 'training, muscle' OR 'occupational therapy'/exp OR 'ergotherapy' OR 'occupation therapy' OR 'occupational therapy' OR 'home rehabilitation'/exp/mj OR 'home rehabilitation' OR 'rehabilitation robot'/exp OR 'brain computer interface' OR 'muscle computer interface' OR 'rehabilitation robot' OR 'robot-assisted rehabilitation' OR 'robotic rehabilitation device' OR 'robot'/exp/mj) AND ('cost'/exp OR 'cost' OR 'cost allocation' OR 'cost sharing' OR 'costs and cost analysis' OR 'deductibles and coinsurance' OR 'cost minimization analysis'/exp OR 'cost benefit analysis'/exp OR 'cost utility analysis'/exp OR 'cost utility' OR 'cost utility analysis' OR 'societal cost'/exp OR 'societal cost' OR 'cost effectiveness analysis'/exp/mj OR 'cost effectiveness' OR 'cost effectiveness analysis' OR 'cost effectiveness ratio' OR 'cost efficiency analysis' OR 'cost benefit analysis'/exp/mj OR 'cost analysis' OR 'cost benefit' OR 'cost benefit analysis' OR 'cost benefit ratio' OR 'cost-benefit analysis' OR 'cost minimisation' OR 'cost minimisation analysis' OR 'cost of illness'/exp/mj OR 'cost of illness' OR 'cost of illness analysis' OR 'economic aspects of illness' OR 'cost consequence analysis'/exp/mj OR 'low cost' OR 'low-cost' OR affordable OR affordability OR inexpensive OR cheap OR cheaper OR economical OR 'budget friendly' OR 'budget-friendly' OR 'cost saving' OR 'cost-saving' OR 'cost savings' OR 'cost containment'/exp OR 'cost containment' OR 'cost reduction'/exp OR 'cost reduction' OR 'cost reductions' OR 'reduced cost' OR 'lower cost' OR 'resource limited' OR 'resource-limited' OR 'low resource' OR 'low-resource' OR 'resource poor' OR 'resource-poor') | |  |  |  |  | 969 |
| Updated #2 | #1 AND ([article]/lim OR [review]/lim) AND ([adult]/lim OR [young adult]/lim OR [middle aged]/lim OR [aged]/lim OR [very elderly]/lim) AND [english]/lim | |  |  |  |  | 248 |
| IEEE Xplore | |  | |  |  |  |  |
| Old #1 | ((( Full Text & Metadata :Cardiovascular) OR ( Full Text & Metadata :Stroke)) AND ( Full Text & Metadata :Rehabilitation ) AND (( All Metadata :Cost) OR ( All Metadata :Cost-Effective))) in command search | | 1294 | 1533 | 1850 | 2214 |  |
| Old #2 | #1 and Filters Applied: Journals | | 299 | 373 | 470 | 561 |  |
| Updated #1 | ((( Full Text & Metadata :Cardiovascular) OR ( Full Text & Metadata :Stroke)) AND ( Full Text & Metadata :Rehabilitation ) AND (( All Metadata :Cost) OR ( All Metadata :Cost-Effective)OR ( All Metadata :Cost-Effectiveness) OR ( All Metadata :Cost Effectiveness) OR ( All Metadata :Economic Evaluation) OR ( All Metadata :Cost Analysis) OR ( All Metadata :Cost-Benefit) OR ( All Metadata :Cost Benefit) OR ( All Metadata :Cost-Utility) OR ( All Metadata :Cost Utility) OR ( All Metadata :Cost-Minimization) OR ( All Metadata :Cost Minimization) OR ( All Metadata :Cost-Consequence) OR ( All Metadata :Cost Consequence) OR ( All Metadata :Health Economics) OR ( All Metadata :Economic Burden) OR ( All Metadata :Low-Cost) OR ( All Metadata :Low Cost) OR ( All Metadata :Affordable) OR ( All Metadata :Affordability) OR ( All Metadata :Inexpensive) OR ( All Metadata :Cheap) OR ( All Metadata :Cheaper) OR ( All Metadata :Economical) OR ( All Metadata :Cost Saving) OR ( All Metadata :Cost-Saving) OR ( All Metadata :Cost Savings) OR ( All Metadata :Reduced Cost) OR ( All Metadata :Lower Cost))) | |  |  |  |  | 2668 |
| Updated #2 | #1 and Filters Applied: Journals | |  |  |  |  | 682 |
| Registries |  | |  |  |  |  |  |
| [Cochrane Reviews (Central)](https://www.cochranelibrary.com/advanced-search) | stroke in All Text AND rehabilitation in All Text AND technologies in All Text - in Cochrane Reviews with 'Stroke' in Cochrane Groups (Word variations have been searched) | | 39 | 43 | 42 |  | 40 |
| [CEA](https://cevr.shinyapps.io/LeagueTables/) | Ischemic Stroke/ Hemorrhagic stroke | | 8 | 8 | 8 |  | 8 |
| [INAHTA](https://database.inahta.org/search?limit=&terms=Stroke+and+Rehabilitation+and+Technology&client=user) | Stroke and Rehabilitation and Technology | | 33 | 34 | 36 |  | 41 |

| Appendix Table 2. CHEERS 2022 Items Reported in Health-Economic Analyses Across Settings | | | | | | | | | | | | | | | | | | | | |
| --- | --- | --- | --- | --- | --- | --- | --- | --- | --- | --- | --- | --- | --- | --- | --- | --- | --- | --- | --- | --- |
|  | **Laboratory Settings** |  | **Clinical Settings** | | | | | | | | | | | **Home Settings** | | | | | |  |
| CHEERS 2022 items |  |  | |  |  |  |  |  |  |  |  |  |  |  |  |  |  |  |  |  |
|  | Vourvopoulos A. *et al. 2016* | Bustamante Valles K. *et al. 2016* | | Hesse S. *et al. 2014* | Pila O. *et al. 2022* | McCabe J. *et al. 2015* | Vanoglio F. *et al. 2016* | Fernandez-Garcia C. *et al. 2021* | Wagner T.H. et al. 2011 *(*Lo. A. et al. 2010) | Rémy-Néris O. *et al. 2021* | Masiero S. et al 2014 | Shankar R. et al., 2025 | Islam M.K. *et al. 2019* | Adie K. *et al. 2017* | Veras M. *et al. 2020* | Housley S.N. *et al. 2016* | Smyada K.E. et al. 2024 | Aguirre_Olliger G. et al. 2024 | Lloréns R. *et al. 2014* | Fusari G. et al., 2025 |
| 1 | 1 | NR | | 637 | 1 | NR | NR | 1 | 1 | NR | 1 | 1 | 1 | 1 | 98 | 1 | 1 | NR | 1 | NR |
| 2 | 1 | 1 | | 637 | 1 | 981 | 351 | 1 | 2 | 1938 | NR | 1 | 1 | 1 | 98 | 1 | 1 | 1 | 4-5 | 1 |
| 3 | 1-2 | 2-3 | | 638 | 2 | 981-82 | 352 | 1 | 2-3 | 1938-39 | 1-2 | 1-2 | 1-2 | 2 | 98-99 | 2-3 | 1-2 | 3-4 | 6-7 | 2-3 |
| 4 | 2 | 6-7 | | 641 | 3 | NR | 354 | 2 | 3/S-pg3 | 1940 | 2-3 | 3-4 | 2-3 | 4 | 100 | 6 | 3 | 8-10 | NR | 4-5 |
| 5 | 2 | 4 | | 638 | 2 | 982 | 352-53 | 2 | 3 | 1939 | 2 | 2-3 | 2 | 2-3 | 99 | 3-4 | 4 | 5 | 10 | 3-4 |
| 6 | 2-3 | 3 | | 638 | 2-3 | 981, 987 | 352-54 | 2-3 | 3/S-pg2 | 1939 | 2 | 1-2 | 2-3 | 2 | 100 | 4 | 3-4 | 5 | 8-9 | 4 |
| 7 | 3 | 4 | | 639-40 | 2 | 982 | 353 | 2 | 2-3 | 1939-40 | 2 | 3 | 2 | 2-3 | 99 | 4-5 | 4 | 8 | 8-9 | 4 |
| 8 | NR | NR | | NR | NR | NR | NR | 2 | S-pg3 | 1940 | 2 | 4 | 2 | NR | 99 | 4 | 3 | 8 | NR | 4 |
| 9 | NR | 7 | | 642 | 3 | NR | 356 | 4 | 3/S-pg4 | 1940 | 2 | 4 | 2 | 3 | 100 | 6 | 3 | 8 | NR | NR |
| 10 | NR | NR | | NR | NR | NR | NR | 3 | S-pg6 | S-pg3 | NR | NR | NR | NR | 100 | NR | NR | NR | NR | NR |
| 11 | 3-4 | 6 | | 640 | 2-3 | 982-83 | 353-54 | 3 | 3/ S-pg2 | 1940 | 2 | 3-4 | 2 | 3 | NR | 5-6 | 5 | 9 | 10 | 5-6 |
| 12 | 3-4 | 6 | | 640-41 | 2-3 | 983-84 | 353-54 | 3 | 3/ S-pg2 | 1940-41/ S-pg3 | 2 | 4 | NR | 3 | NR | 5-6 | 5 | 9 | 9 | 5-6 |
| 13 | 3-4 | 6 | | 639 | 2 | 983-84 | 352-54 | 3-4 | 3/ S-pg2 | 1939/ S-pg1 | 2 | 4 | 2-3 | 3 | 99 | 4-6 | 5 | 8-9 | 10 | 5-6 |
| 14 | 3 | 6-7 | | 641 | 3 | 987 | 354 | 2-3 | 3/ S-pg2 | S-pg3 | 2-3 | 4,5,S | 2 | 4 | 100 | 6 | 5 | 8,18,22 | 10, 29 | 13 |
| 15 | NR | 7 | | NR | NR | NR | NR | 3 | 3/ S-pg3 | 1940-41/ S-pg4 | NR | 4 | 4 | NR | 100 | 6 | 5 | 8, 18 | 10, 29 | 14-15 |
| 16 | 5 | 6-7 | | 642 | 3 | NR | 354, 356 | NA | NA | NA | 2-3 | 4,S | 2-3 | NA | 100 | 6 | 4-5 | 18,22 | NR | NA |
| 17 | NR | 7 | | 641 | 3 | 983-84 | 354 | 3-4 | 3/ S-pg3 | 1940-41 | NR | 4-5,S | NR | 4 | NR | 6-7 | 3 | 8 | NR | 6 |
| 18 | NR | NR | | NR | NR | 984 | NR | 4 | 3/ S-pg3-4 | NR | NR | 2-3,5 | 3 | NR | NR | NR | NR | 9 | NR | NR |
| 19 | NR | NR | | NR | NR | NR | NR | 4 | 3/ S-pg3-4 | NR | NR | NR | NR | NR | NR | NR | NR | NR | NR | NR |
| 20 | NR | NR | | NR | NR | NR | NR | 3-4 | 3/ S-pg4 | 1941 | NR | 4-6 | 2-3 | 4 | NR | NR | 5 | NR | NR | NR |
| 21 | 4 | NR | | NR | NR | NR | NR | 70-91, *Rodgers et al.* 2022) | NR | 1940 | NR | 5-6 | NR | 3 | NR | 6 | *Smyada et al. 2023* | 7 | 9-10 | NR |
| 22 | NR | 9-10 | | 641 | NR | NR | 356-57 | 4-9 | 3-4/ S-pg4 | 1944/S-pg4 | 2-3 | 4,5,S | 3 | 4-8 | NR | 13-14,21-22 | 6-7 | 13,15 | 12-13, 29 | 13-15 |
| 23 | 5 | 9-11 | | 641-45 | 4-5 | 984-88 | 355-57 | 4-5 | 3-4/ S-pg4-5 | 1944 |  | 5-7 | 4-5 | 8 | 100-102 | 13-14,21- 25 | 6-7 | 15 | 12-13, 29 | 13-15 |
| 24 | NR | NR | | NR | NR | NR | NR | 9 | S-pg5 | NR | NR | 5-8 | NR | 8 | NR | NR | 6-7 | NR | NR | NR |
| 25 | 5 | NR | | NR | NR | NR | NR | 70-91, *Rodgers et al. 2022* | NR | 1944 | NR | 4,8 | NR | NR | NR | 12,24 | *Smyada et al. 2023* | 12-13 | 15 | NR |
| 26 | 5-6 | 10-13 | | 645-46 | 3-5 | 988 | 358 | 10-11 | 4/ S-pg6 | 1944-46 | 4 | 7-9 | 4 | 8-11 | 103 | 13-14,20, 25 | 7-8 | 17-18 | 13-16 | 15-17 |
| 27 | 6 | 13 | | 646 | 6 | 981 | 359 | 11 | 4 | 1946 | 5 | 9 | 1 | 12 | 104 | 15 | 9 | 28 | 2 | 17 |
| 28 | NR | 14 | | 646 | 7 | 981 | 359 | 11 | 1 | 1946 | 5 | 9 | 5 | 12 | 104 | NR | 9 | 28 | NR | 18 |
| RI (n) | 17 | 19 | | 19 | 18 | 15 | 18 | 27 | 25 | 23 | 18 | 26 | 21 | 21 | 18 | 22 | 25 | 23 | 17 | 18 |
| NA (n) | 0 | 0 | | 0 | 0 | 0 | 0 | 1 | 1 | 1 | 0 | 0 | 0 | 1 | 0 | 0 | 0 | 0 | 0 | 1 |
| Total (%) Reported items | 60.7% | 67.9% | | 67.9% | 64.3% | 53.6% | 64.3% | 96.4% | 89.3% | 82.1% | 64.3% | 92.8% | 75.0% | 75.0% | 64.3% | 78.6% | 89.3% | 82.1% | 60.7% | 64% |
| The numbers correspond to the page numbers of the included articles. ^a^ NA=Not applicable, Pg=Page, RI=Reported items, S=Supplement. | | | | | | | | | | | | | | | | | | | | |

**Appendix Table 3. Details of Health-Economic Analyses in Clinical Settings**

| Authors, year | Cost-analysis’ type | Intervention | | | | | Comparator | | Economic outcomes | | | | | | |
| --- | --- | --- | --- | --- | --- | --- | --- | --- | --- | --- | --- | --- | --- | --- | --- |
|  |  |  |  | **TAR** |  |  | **CT** | **CT** |  | **TAR** |  | **TAR vs CT** | **CT** | **TAR** | **TAR vs CT** |
|  |  | **Devices** | **Session** | **Added CT**  **Session** | **T/P of**  **TAR** | **Relative T/P of TAR to Comparator** |  | **Cost Per CT Session** | **Retail Price (italic) or Total Production Cost of TAR devices** | **Total Overhead (Including Maintenance)Cost for TAR Devices** | **Cost Per TAR Session** | **Difference in Cost Per Session** | **Health and Social Service Use Cost** | **Health and Social Service Use Cost** | **Difference in Cost of Health and Social Service Use Cost, Including Intervention Cost** |
|  |  |  | **Min/ Session** | **Min/ Session** |  |  | **Min** | **US$** | **US$** | **%** | **US$** | **%** | **US$** | **US$** | **%** |
|  |  |  |  |  |  |  |  | **a** |  |  | **b** | **(a-b)/a** | **c** | **d** | **(c-d)/c** |
| Bustamante Valles K. et al., 2016  [64] | CM | Robot gym  *(1 end-effector robot,2 electromechanical motor-assisted ergometer, 1 computer-based cognitive rehabilitation device, 2 FES devices)* | 120  *(Standalone TAR)* | 0 | 0.25  *(One therapist can supervise 6 stroke survivor, however in the study only 4 stations were used)* | 0.25 | 120 | 19.2  *(Therapist annual salary is US$ 19612 and needed equipment cost is US$ 7000. One therapist supervises 4 stroke survivors per 8 working hours in a day. Over 278 days, or annually a therapist treats a total of 1112 stroke survivors.)* | *36048*  *(Reported)*  *(6 devices, life span NR)* | 25  *(Reported)*  *(overhead cost for 2 years, annually US$ 9012, total US$ 18024)* | 7  *(Assumption - the gym to be used by 24 stroke survivors per day, 6672 stroke survivors annually over 278 days)* | 64 | NR | NR | NR |
| Hesse S. et al., 2014 [65] | CM | Robot gym  *(1 end-effector robot, 1 computerised arm trainer, 2 mechanical arm trainers, 2 electromechanical finger trainers)* | 30  *(Added to CT)* | 30 | 0.29  *(One therapist supervised 3-4 stroke survivors)* | 0.65 | 60 | 13.3*  (*CT reported to be used by 15 stroke survivors per day, total of 255 days; equal to total 3825 stroke survivors annually. Total cost of the CT would be US$ 51131.9*, including annual salary of senior therapist US$ 46018.7* and 10 % overhead annual cost of US$ 5113.2*)* | NR directly    *47812**  *(Estimated)*  *(6 devices, life span of 4 years)*  *(Reported total gym cost US$ 63749*, which included 25% overhead cost. Estimated total annualised overhead cost of US$ 15937*)* | 25  *(Reported)*  *(Including energy, consumables, maintenance)* | 5.5*  (Standalone TAR)  (*The gym to be used by 35 stroke survivors per day over 255 days, in total by 8925 stroke survivors annually; Total cost of the gym US$ 49139.7*, includes salary of assistant therapist US$ 33202.5* and annual equipment cost US$ 15937.2**) | 59 | - | - | - |
| Pila O. et al., 2022  [66] | CM (Assumed) | End-effector robot  (*Assumed 2 end-effector robots*) | 60  *(added high-dose TAR to CT)* | 60 | 0.5 | 0.75  (*Assumed*  *60 min of adjunct TAR plus 60 min of CT vs 120 min CT)* | 120  (*Assumed CT, with T/P of 1)* | NR directly  81.2*  *(Estimated)*  *(Assumed as reported*  *120 min of CT, each 60 min session cost US$ 40.6*)*  (*CT to be used by 7 stroke survivors per day over 235 days a year, in total 1645 sessions; total annual salary of therapist US$ 66806.4*)* | *107903.9**  *(Reported)*  *(The device life span 7 years; The device usage 7 sessions per day, annually for 235 days, in total, annually for 1645 sessions; Robot’s annual total cost US$ 19268.5)* | NR directly  25  *(Estimated)*  *(Reported total operating cost of US$ 26,977*. Estimated*  *total operating cost at 25% of total retail price over the 7 years)* | NR directly  82*  *(Estimated)*  *(Assumed*  *60 min of adjunct TAR cost US$ 41.4* and 60 min of CT cost US$ 40.6*)*  *(Total cost of the TAR plus CT was US$ 2542.2* for 31 sessions, each of 120 min)* | NR directly  -1  *(Estimated)* | - | - | - |
| McCabe J. et al., 2015  [67] | CM | End-effector robot | 90  *(added to CT)* | 210 | 0.33  *(One therapist supervised three different interventions)* | 0.8 | 300 | NR directly  76.2  *(Estimated)*  *(Total cost of CT was US$ 4570 for 60 sessions)* | *89000*  *(The device’s life span 5 years)* | NR directly  36  *(Estimated)*  *(Reported, annual overhead cost US$ 8000; Estimated total overhead cost as 36% of total retail price for 4 years of use, over 5 years of lifespan)* | NR directly  94.8  *(Estimated)*  *(TAR and CT, total cost was US$ 5686 for 60 session)* | -24  *(Estimated)* |  |  | - |
| Vanoglio F. et al., 2017 [68] | CM | Exoskeleton | 40  (*Standalone TAR)* | 0 | 0.3  *(One therapist was present 24/40 minutes during 3 sessions, 11/40 minutes during other 27 sessions)* | 0.3 | 40 | NR directly  17.8*  *(Estimated)*  *(Total cost of CT was US$ 532.6*, per minute therapist cost US$ 0.444, total of 40 min of 30 sessions)* | NR directly  *26630.4**  *(Estimated)*  *(The device’s life span 5 years, total cost US$ 33,288, including 20% annual overhead cost)* | 20  *(Reported)*  *(Reported total cost of the device was US$ 33288, which included 20% of annual overhead cost. The amount of overhead yearly cost is estimated at US$ 1664.5* during 4 years of use, over 5 years of lifespan)* | NR directly  8.8*  *(Estimated)*  *(Total cost of TAR was US$ 263.2*; The device to be used by 8 patient per day, for a cycle of 30 days, in total for 250 days, 67 patient per year, 335 stroke survivors in total over 5 years; per minute therapist cost US$ 0.444*, total of 24 min in the first 3 sessions and 11 minutes in the rest of 27 sessions, plus device cost US$ 99.5*)* | 51  *(Estimated)* | - | - | - |
| Fernandez-Garcia C. et al., 2021 [69, 93, 94] | CU | Robot gym  *(2 end-effector robots and 1 attachment*) | 45 | 45  (*Usual therapy)* | 1 | 1 | 45  *(CT - enhanced therapy group)*  *&*  45  (*Usual therapy)* | NR directly  51.9*  *(Estimated)*  *(Total intervention cost US$ 1869.5 divided over N of sessions of 36)* | *1336300**  *(2 devices, with a life span of 5 years. The retail price was annually discounted by 3.5%)* | NR directly  6  *(Estimated)*  *(Reported annual maintenance costs US$ 21693.5. Estimated total maintenance cost as 6% of total retail price for 4 years)* | NR directly  106.6*  *(Estimated)*  *(Total intervention cost US$ 3837.9 divided over N of sessions of 36)* | -105  *(Estimated)* | *5947.9*  *(at 6 months)* | 7198.6  *(at 6 months - enhanced therapy group)* | -21 |
| Lo A.C. et al. 2010 [70, 95] | CU | Robot gym  *(3 end-effector robots)* | 60  *(Standalone TAR)* | 0 | 0.25 | 0.25 | 60  *(CT–intensive comparison therapy group)* | 218.0  *(The therapist cost for 60 minutes was US$ 218 - intensive comparison therapy group)* | *230750*  (*3 devices; with a life span of 5 years)* | NR directly  83  *(Estimated)*  *(Reported annual gym maintenance cost 6.5% for 4 years; annual cost of financing 6.015% for 4 years and total facility overhead cost 33% for 5 years. Estimated total overhead cost as 83% of total retail price for 5 years)* | 140.0  *(TAR per session cost US$ 20 and therapist cost US$ 120 for 15 minutes. The gym to be*  *used for 86 sessions per week, a total of 50 weeks annually, and 4300 sessions.)* | 36 | 12364  *(9 months)* | 12679  *(9 months)* | -3 |
| Masiero S. et al. 2014 [74, 96, 97] | CM | End-effector robot  **(***assumed 4 devices in use)* | 40  *(added to CT)* | 80 | 0.5  *(average T/P - scenario when therapist supervision reduces weekly from 30 min/per session in the first week to 7min/per session in the fourth week)* | 0.84 | 120 | 24.9*  *(Therapist’s annual salary of US$ 43880. Annually one therapist works 8 hours daily, over 220 days, total of 1760 hours)* | *66405**  *(Reported)*  *(1 device, life span of 5 years. Effective annual hours in use 2080)* | 32  *(Reported)*  *(Annually US$ 5312.4 over 4 years)* | 21.4*  *(average cost per hour of TAR session - scenario when therapist supervision halves weekly from 30 min/per session in the first week (hourly cost US$ 27,13 in the first phase) to 7min/per session in the fourth week (average hourly cost US$ 15,7 in the second phase))* | 14  *(Estimated)* | - | - | - |
| Shankar R. et al., 2025 [75] | CU | Exoskeleton | 30 min *(added to CT)* | 15-30 | 1 | 1 | 45-60 | NR directly  48.7  *(Estimated)*  *(equipment and part time TA assistant cost US$ 14.63 /session 7.14 +consumables cost per session US$ 2.22 +US$ 44.,4 therapist cost per session)* | *209642*  *(The device’s life span 8 years)* | 35  *(Estimated)*  (*US$ 10360* *annualised over 7 years*) | NR directly  109  *(Estimated)*  *(equipment cost for 6 months US$ 2560.59 / 42 patients + +consumables cost per session US$ 3.7 + US$ 44.4 therapist cost per hour)* | -124  *(Estimated)* | NR | NR | NR |
| Islam M. K. et al., 2019 [77, 98] | CM | VR  & wearable sensors | 60  *(added to CT)* | 120 | 0.25 | 0.75 | 180 | NR directly  36.7  *(Estimated)*  *(Scenario 1: combination of 20 min TAR and 40 min CT: Total cost of CT for 80 stroke survivors equal to US$ 44055)* | *37720*  *(The device’s life span 5 years, source-the device manufacturer)* | NR | NR directly  34.1  *(Estimated)*  *(Scenario 1: combination of 20 min TAR and 40 min CT:*  *Total cost of TAR for 40 stroke survivors equal to US$ 20446)* | 7  *(Estimated)* |  |  | - |
| Rémy-Néris O. et al., 2021 [78] | CU | Exoskeleton *(gravity supported mechanism)*  &  VR | 60  *(added to CT)* | 90 | 0.1  *(therapist attended 4 sessions out of 40 sessions, each of 30 min)* | 1.07 | 60 (SR),  90 (CT) | NR directly  40.0  *(Estimated)*  *(Assumed cost of 60 min CT. Each session of*  *30 min of inpatient CT cost US$ 17 and outpatient CT cost US$ 23, average cost of 30 min CT US20)* | *73220*  *(The device’s life span 5 years)* | NR directly  26  *(Estimated)*  *(Reported overhead cost as annual*  *6.5%. Estimated total overhead cost as 26% of total retail price for 4 years)* | NR directly  94.6*  *(Estimated)*  *(reported per patient device cost US$ 1894.7*/ 20 sessions)* | -136.5  *(Estimated)* | 60252*  *(12 months)* | 60239.6*  *(at 12 months)* | 0.02 |

Appendix Table 3:

*Costs were converted from the original currency to US$:

1. *Hesse S. et al., 2014 , Euro 1=US$ 1.3281 in 2013*
2. *Pila O. et al., 2022 , Euro 1=US$ 1.1194 in 2019*
3. *Vanoglio F. et al., 2017 , Euro 1=US$ 1.1096 in 2016*
4. *Rémy-Néris O. et al., 2021 , Euro 1=US$ 1.1847 in 2021*
5. *Fernandez-Garcia C. et al., 2021 , GBP 1=US$ 1.3363 in 2018*
6. *Masiero S. et al. 2014 , Euro 1=US$ 1.3281 in 2014*

Retail prices are italicised. All numbers were rounded off to one decimal.

^a^ BCI=Brain-Computer Interface, CT=Conventional therapy, CM=Cost-Minimisation, CU=Cost-Utility, FES=Functional Electrical Stimulation; Min=minutes, MCI=Muscle Computer Interface, NA= Not Applicable, NR=Not Reported, rT/P= relative Therapist-to-Patient ratio of combined therapy (TAR and CT) to comparator (CT in a clinical setting), TAR= Stroke Technology Assisted Rehabilitation, SR=Self-Rehabilitation T/P= Therapist-to-Patient ratio, VR=Virtual Reality

**Appendix Table 4. Details of Health-Economic Analyses in Home Settings**

| **Authors, Year** | **Type of Cost Analysis** | **Intervention** | | | | | | **Comparator** | | |  | **Economic outcomes** | | | | | | | | |  |
| --- | --- | --- | --- | --- | --- | --- | --- | --- | --- | --- | --- | --- | --- | --- | --- | --- | --- | --- | --- | --- | --- |
|  |  | **TAR** | | | | | | **CT/Other** | | | **CT/Other** | **TAR** | **TAR** | **CT/Other** | **TAR vs CT/Other** | **TAR** | **TAR vs CT/Other** | **CT/Other** | **TAR** | **TAR vs CT/Other** | |
|  |  | **Devices (n)** | **Prescribed**  **TAR** | | **Used TAR** | **Add**  **CT**  **Session to TAR** | **Therapist Time in TAR** | **Prescribed CT** | | **Used CT** | **Total Cost of Comparator** | **Retail Price (italic) or Total Production Cost of TAR Devices** | **Total Cost of TAR Intervention** | **Transportation Cost of Comparator** | **Difference in Total Cost** | **Transportation Cost of**  **Intervention** | **Difference in Transportation Cost** | **Health and Social Service Use Cost** | **Health and Social Service Use Cost** | **Difference in Cost of Post-intervention** **Health and Social Service Use, Including Intervention Cost** | |
|  |  |  | **N of Sessions** | **Min/ Session** | **Total Min** | **Min/ Session** | **Total Min** | **Session** | **Min/ Session** | **Total Min** | **US$** | **US$** | **US$** | **%** | **US$** | **US$** | **%** | **US$** | **US$** | **%** | |
|  |  |  |  |  |  |  |  |  |  |  | **a** |  | **b** | **(a-b)/a** | **c** | **d** | **(d-c)/d** | **e** | **f** | **(e-f)/e** | |
| Veras M. et al., 2020  [81] | NA | VR  *(4 devices)* | 20 | 30 | - | 0 | 75 | 20 | 30  *(SR)* | - | - | 6071*  *(4 units cost of US$ 6071*, a life span of 4-5 years)* | 376.6*  *(Annualized and discounted by 3%, total cost was US$ 15066.1* for 40 stroke survivors per year)* | - | - | 26.5*  *(a sum of cost incurred by installation and removal, as well as hardware assistance mileages (km).)* | - | - |  | - | |
| Lloréns R. et al., 2015  [80] | CM | VR | 20 | 45 | - | - | 98 | 20 | 45  *(CT in clinic)*  &  Usual therapy  *(amount NR)* | NR | 1490.2  *(Total cost of CT 34 sessions cost)* | 800 | 835.6 | 44 | 1308 | 0 | 100 | - | - | - | |
| Adie K. et al., 2017  [82] | CU | VR | 30 | 45 | 1020 | *Amount NR*  *(Usual therapy)* | - | 30 | 45  (SR)  &  CT *(amount NR)* | 998  (SR) &  CT *(amount NR)* | 0 | - | 175.1* | NA | - | - | - | 1141.9 *  *(6 months)* | 1730*  *(at 6 months)* | -51.5 | |
| Smayda K. E.et al., 2024 [86, 99, 100]**, *** | BIA  ** | Wearable sensors & auditory feedback | 24  *(TAR–Assumed 2 months, 3 sessions weekly)* | 30 | - | 0 | NR | 24  *(Assumed)* | 30  *(Assumed –CT in clinic)* | - | 1396.3  *(per patient annually, cost per 30 min 75US$)* | NR  *(rent fees for 5 weeks 1,450 US)* | 2320  *(rent fees for 8 weeks)* | -66 | - | - | - | 9795.8  *(12 months)* | 8377.2  *(12 months)* | 14.5 | |
| Housley S.N. et al., 2016 [87] | CM | End effector robots & VR  *(2 devices)* | 90 | 60-120 | 2763.6  (*Reported completed average 30.6/106 sessions (29.3%), 90.6/120 min/session (75.5%), total 2763.6//12,720 min (21.7%). 135.4% increase of exposure compared to CT)* | - | - | 14.7  (*Reported- total CT sessions, based on reports of previous use of CT, 1.2 sessions weekly or 2-4 times per month)* | 60  (*Reported –assumed CT in clinic)* | - | 1563.39  *(Reported therapist monthly cost US$ 521.13, over 3 months is 1563.39)* | NR  *(Reported* *average monthly cost US$ 68.45, monthly maintenance cost of a device US$ 99.92)* | 761.2  (*Reported* *average monthly cost US$ 68.45, monthly maintenance cost of a device US$ 99.92, and therapist cost US$ 85.37. Total monthly cost US$ 253.74 for 3 months*) | 51.3 | 2056.7  *(Reported monthly transit cost of US$ 685.5 for 3 months)* | 506.9  *(Reported monthly transit cost of US$169 for 3 months)* | 75 | NA | NA | NA | |
| Aguirre-Ollinger G. et al., 2024 [88, 101] | CE&  BIA | End effector robot & VR | 30 | 60-120 | 2025.1  *(average completed 26.3/30 sessions (87.7%), 77/120 min/session (64.1%), total 2025.1/3600 min (56.3%))* | 60  *(CT in clinic -*  *Usual therapy*  *)* | 550-640  *(remote monitoring 10 mins for 10 times*  *)* | 28  *(assumes - CT in clinic)* | 60  *(CT in clinic)*  &  60  *(Usual therapy*  *)* | - | 2314.5 * | NR | 1753* | 24.3 | 264.7* | 66.8* | 75 | - | - | - | |
| Fusari G. et al., 2025 [79] | CU | Wearable sensor | NA | NA | NR | NR  *(Usual therapy*  *)* | NR | NR | NR  *(Usual therapy)* | NR | 0 | *4287**  *(maintenance cost of a device US$ 494.7)* | 2017.1* | NA | NR | NR | - | 18553.3* | 9680.3* | 48 | |

Appendix Table 4:

*Costs were converted from the original currency to US$. All numbers are rounded off to one decimal.

1. *Veras M. et al., 2020, CA$ 1.2791=US$ 1 in 2015*
2. *Adie K. et al., 2017, GBP 1= US$ 1.5642 in 2013*
3. *Aguirre-Ollinger et al., 2024, SG$ 1.3787=US$ 1 in 2021*
4. *Fusari G. et al., 2025, GBP 1=US$ 1.3192 in Jan. 2025*

**Study outcomes derived from BIA.

^a^BCI=Brain-Computer Interface, BIA= Budget Impact Analysis, CE=Cost-Effectiveness, CM=Cost-Minimisation, CU=Cost-Utility, CT=Conventional therapy, MCI=Muscle Computer Interface, NA=Not Applicable, NR=Not Reported, TAR= Stroke Technology Assisted Rehabilitation, SR=Self Rehabilitation, VR=Virtual Reality
